# Global invasion genetics of two parasitic copepods infecting marine bivalves

**DOI:** 10.1038/s41598-019-48928-1

**Published:** 2019-09-04

**Authors:** Marieke E. Feis, M. Anouk Goedknegt, Isabelle Arzul, Anne Chenuil, Onno den Boon, Leo Gottschalck, Yusuke Kondo, Susumu Ohtsuka, Lisa N. S. Shama, David W. Thieltges, K. Mathias Wegner, Pieternella C. Luttikhuizen

**Affiliations:** 1 Coastal Ecology Section, Alfred Wegener Institute, Helmholtz Centre for Polar and Marine Research, Wadden Sea Station Sylt, Hafenstraße 43, 25992 List/Sylt, Germany; 2 Graduate School of Integrated Science for Life, Hiroshima University, Takehara, Hiroshima 725-0024 Japan; 3 NIOZ Royal Netherlands Institute for Sea Research, Department of Coastal Systems, and Utrecht University, P.O. Box 59, 1790 AB Den Burg, Texel, the Netherlands; 4 Ifremer, Laboratoire de Genetique et Pathologie des Mollusques Marins, Avenue Mus Loup, F-17390 La Tremblade, France; 5 Aix Marseille Univ, Univ Avignon, CNRS, IRD, IMBE, Marseille, France; 6 Present Address: Sorbonne Université, CNRS, Station Biologique de Roscoff, Laboratoire Adaptation et Diversité en Milieu Marin, DyDIV, UMR 7144, 29688 Roscoff, France; 7 Present Address: Université de Bordeaux, UMR 5805 EPOC, Station Marine d’Arcachon, 2 Rue du Professeur Jolyet, 33120 Arcachon, France

**Keywords:** Invasive species, Genetic variation, Zoology

## Abstract

Invasive species, and especially invasive parasites, represent excellent models to study ecological and evolutionary mechanisms in the wild. To understand these processes, it is crucial to obtain more knowledge on the native range, invasion routes and invasion history of invasive parasites. We investigated the consecutive invasions of two parasitic copepods (*Mytilicola intestinalis* and *Mytilicola* *orientalis*) by combining an extensive literature survey covering the reported putative native regions and the present-day invaded regions with a global phylogeography of both species. The population genetic analyses based on partial COI sequences revealed significant population differentiation for *M*. *orientalis* within the native region in Japan, while introduced populations in North America and Europe could not be distinguished from the native ones. Thus, *M*. *orientalis*’ invasion history resembles the genetic structure and recent spread of its principal host, the Pacific oyster, *Crassostrea gigas*, while *M. intestinalis* lacks population genetic structure and has an overall low genetic diversity. Therefore, the native origin of *M*. *intestinalis* remains unclear. With this study, we demonstrate that even highly related and biologically similar invasive species can differ in their invasion genetics. From this, we conclude that extrapolating invasion genetics dynamics from related invasive taxa may not always be possible.

## Introduction

Biological invasions (whether recognized or cryptic) can alter ecosystem functioning and services^1^, and can have ecological and evolutionary impacts on native communities and ecosystems (e.g.^2–5^). Invasive species can also be parasites, which may be introduced with or without their hosts. Parasite invasions can have wide-ranging effects on host-parasite interactions with strong ecological and evolutionary implications^6^. To examine the effects of invasive parasites, a detailed understanding of their origins and invasion pathways is important. To this extent, the traditional approach of using literature records can be fruitfully complemented by population genetic analyses. Phylogeographic studies of species in their putative native and invaded regions (“invasion genetics studies”)^7^ can provide more detail to clarify the origin, invasion routes, population genetic structure and genetic diversity of invasive parasites^8^. However, invasive parasites are greatly understudied in this respect, as well as with regard to their ecological and evolutionary impacts^9^.

Two exceptions are the consecutive invasions of the parasitic copepods *Mytilicola intestinalis* Steuer 1902 and *Mytilicola orientalis* Mori 1935 along the European Atlantic shores. Both of these natural experiments have been proven to be particularly useful to study their ecological consequences and coevolution with the blue mussel, *Mytilus edulis*, one of their host species. *Mytilicola intestinalis* invaded European waters first in the 1930s presumably through ship hull fouling and relaying of infected mussels (e.g.^10–12^). Its invasion into the western European Wadden Sea was recently used as a model system to study host-parasite coevolution in the wild^13,14^. Cross-infections of hosts and parasites from separate invasion fronts where the parasite had invaded both locations at a similar time (i.e., similar lengths of coevolutionary time) showed that parasite invasion led to different evolutionary trajectories, with differences in the parasite’s infectivity and the host’s resistance and tolerance^13^. These trajectories were characterized by specific molecular interactions between host and parasite^14^. *Mytilicola orientalis* was co-introduced with the invasive Pacific oyster, *Crassostrea gigas*, through aquaculture transports^15,16^, and now also infects a suite of native hosts, including blue mussels, *M*. *edulis*^16–18^. Thus, the native host, *M*. *edulis*, is now shared between *M*. *intestinalis* and *M*. *orientalis*.

The reported invasion histories already suggest substantial differences in the mode of invasion between these two *Mytilicola* species that could, in turn, lead to dissimilarities in ecological effects and evolutionary responses in *M*. *edulis*. However, while the major invasion route of the principal host of *M*. *orientalis*, the Pacific oyster, is known^19,20^, the invasion route of its hitchhiking parasite has yet to be validated. Similarly, the dispersal routes of *Mytilus galloprovincialis* are better known than those of its parasitic copepod *M*. *intestinalis*^21,22^. To fully understand the parasites’ invasion histories and their consequences for ecological and evolutionary processes, the genetic structure in the native ranges and along invasion routes of both parasite species needs to be investigated.

*Mytilicola* is a genus of cyclopoid copepods that have a direct life cycle involving one pelecypod host. Their free-living larval stages are short and have mainly been studied in *M*. *intestinalis* (e.g.^10,23,24^). The duration of the free-living and parasitic larval stages of the other *Mytilicola* species are thought to be similar^25^. The two free-living larval stages, nauplius and metanauplius, last 36 to 48 hours at a laboratory temperature of 18 °C^23^. The next larval stage, the first copepodite, is infective and will search for a suitable host to infect. It may live for 11 to 15 days under laboratory conditions, but it is doubtful whether it lives that long in the field^23^. Once *Mytilicola* has infected a host, its dispersive capacity depends on its bivalve host, which is sessile and does not move across long distances unaided. Therefore, the dispersal capacity of *Mytilicola* was considered to be limited^12,26^.

In this study, we aimed to (1) use the available literature to reconstruct the invasion history of both parasites, and (2) test whether the reported invasion routes could be confirmed by a global phylogeography based on partial cytochrome-*c*-oxidase I (COI) sequences. We specifically wanted to verify their putative areas of origin (i.e. Mediterranean Sea Basins for *M*. *intestinalis* and Japanese seas for *M*. *orientali*s), and determine the genetic diversity and population differentiation between putative native and invaded regions for both invasive *Mytilicola* species.

## Results

### Literature-based invasion history

A search for “*Mytilicola intestinalis*” resulted in 72 hits on the Web of Science/Web of Knowledge (all databases) and 934 hits on Google Scholar (on 15 December 2017). A search for “*Mytilicola orientalis*” resulted in 15 hits on the Web of Science/Web of Knowledge (all databases), and 373 hits on Google Scholar (on 15 December 2017). More literature was found by snowballing (see Methods). Our literature survey led to the inclusion of 173 publications on occurrences of *M*. *intestinalis* (2187 data points including those of this study; reference list in Supplementary Information) and of 47 publications on occurrences of *M*. *orientalis* (416 data points including those of this study; reference list in Supplementary Information). The literature included peer-reviewed publications, fishery leaflets, reports, book sections and dissertations. A total of 41 publications with potentially relevant information could not be read because the full texts were not available online, neither accessible via Dutch and German national libraries, nor by attempts to contact the original authors, and thus, could not be included.

**Figure 1:**
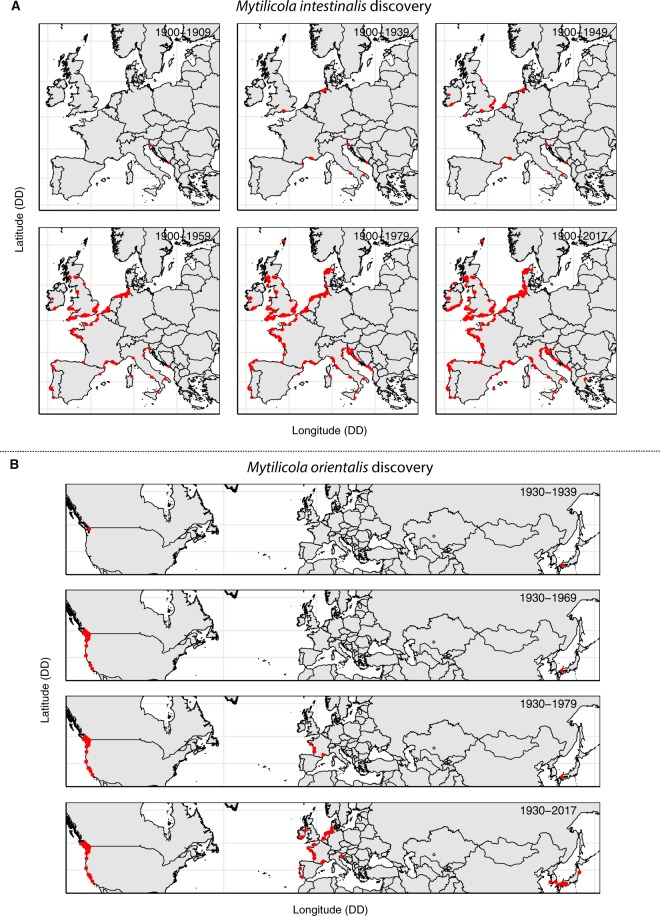
Temporal development of the known distributions and invasions of (**A**) *Mytilicola intestinalis* and (**B**) *Mytilicola orientalis* from time of first observation and species description (i.e., 1902 for *M*. *intestinalis* and 1935 for *M*. *orientalis*) until December 2017. Locations where *Mytilicola* spp. presence has been recorded are indicated with red dots, showing the species distribution, invasion route and spread, as well as research effort through time. For temporal development of *Mytilicola* spp. invasions decade-by-decade, including species absence records, see Supplementary Figs S1 and S2. For animated temporal development of *Mytilicola* spp. year-by-year, see the .gifs in Online Resources 1 and 2. This figure is based on data from this study and from data acquired through our literature surveys. See Supplementary Information for reference lists.

The distribution of *Mytilicola intestinalis* before the 1930s was limited to the Mediterranean Sea Basin, where the parasite was recorded from a few locations near large ports and aquaculture areas (Figs 1A, S1 and Online Resource 1). The hypothesized native region of *M*. *intestinalis* is, therefore, the Mediterranean Sea Basin. In the 1930s, its distribution extended to a few specific locations at the German and English coasts, from which, in the following years, the parasite spread to coasts of neighboring countries. In the late 1940s, the first sightings in the Netherlands and Ireland were reported, followed by the Atlantic coasts of France in the early 1950s. In 1951, researchers collaborated to record the invasion and potential effects of *M*. *intestinalis* along the Atlantic coasts of Europe, which resulted in the publication of a special issue in Revue des Travaux de l’Office Scientifique et Technique des Pêches Maritimes on *M*. *intestinalis*^27^. Ship hull fouling was seen as the main vector of *M*. *intestinalis* introductions, after which further spread was aided by the relaying of mussels (i.e., from the German Wadden Sea to aquaculture areas in Zeeland, the Netherlands). It is, thus, unclear from which Mediterranean basin (or basins) *M*. *intestinalis* was introduced, and whether this happened repeatedly, which might lead to different expectations regarding signatures of bottlenecks and genetic diversity in the introduced region.

The distribution of *M*. *orientalis* in its putative native range has not been the subject of many studies, which is reflected in our literature survey (Figs 1B, S2 and Resource 2). The co-introduction of *M*. *orientalis* with the Pacific oyster in North America was better recorded. Where young Pacific oysters or infected stock were imported, *M*. *orientalis* was also found, but the parasite did not spread quickly to adjacent natural areas^26^. In the 1970s, Pacific oysters from North America were imported into France, and, once more, *M*. *orientalis* was co-introduced across half the globe. In 1993, the species was also found in Ireland in imported oysters from France^28^. There are many gaps in the distribution of *M*. *orientalis* in Europe, which presumably reflect the lack of studies in these areas (Supplementary Fig. S2, Online Resource 2).

### New records and infection levels of *Mytilicola* spp

A total of 5604 Pacific oysters (*Crassostrea gigas*), 2658 Mediterranean mussels (*Mytilus galloprovincialis*), more than 1595 blue mussels (*Mytilus edulis*), 104 bay mussels (*Mytilus trossulus*) and 90 mussels (*Brachidontes pharaonis*) were dissected in this study. Dissections of Pacific oysters and mussels from 20 locations along Mediterranean coasts, 13 locations along Japanese coasts, 7 locations along Pacific American and Canadian coasts, and 21 locations along European Atlantic, North Sea and Baltic coasts resulted in detection of *M*. *intestinalis* and *M*. *orientalis* at 20 and 30 locations, respectively. In Japan, *M*. *orientalis* has only been previously recorded in Hiroshima^29^ and Takehara^30^, both in the Hiroshima Prefecture, and furthermore in Hiwasa, the Tokushima Prefecture^31^ – the other locations, including those outside the Seto Inland Sea are, thus, new records. Also, Tavira (Portugal) is a new location for *M*. *orientalis*. The Mediterranean locations Malinska, Marjan (both Croatia) and Cassis (France) are new records for *M*. *intestinalis*, although Cassis is close to locations where *M*. *intestinalis* was already known from (i.e., Berre Lagoon and Marseille^32,33^).

*Mytilicola intestinalis* was detected in *M*. *galloprovincialis* and *M*. *edulis*. *Mytilicola orientalis* was also detected in these bivalves, but also in *C*. *gigas* and *M*. *trossulus*. Prevalences of *M*. *intestinalis* ranged from 0 to 82.0% in the Mediterranean Sea Basins, the putative native region of *M*. *intestinalis*, and from 0 to 93.8% in the invaded regions (Table 1). Prevalences of *M*. *orientalis* ranged from 0 to 10.6% in Japan, the putative native region of *M*. *orientalis*, and from 0 to 73.8% in the invaded regions (Table 1).

**Table 1:** Parasitic copepods *Mytilicola intestinalis* (MI) and *Mytilicola orientalis* (MO) collected from mussels: *Mytilus galloprovincialis* (Mg), *Mytilus edulis* (Me), *Mytilus trossulus* (Mt) and *Brachidontes pharaonis* (Bp), and from oysters: Pacific oysters *Crassostrea gigas*.

Location	Country	Coordinates	Year	N_hosts_		Prevalence (%)			Mean infection intensity			N_parasites_		*Collectors* and Dissectors
				mussel	oyster	MI _mussel_	MO _mussel_	MO _oyster_	MI _mussel_	MO _mussel_	MO _oyster_	MI	MO	
**Seto Inland Sea**, **Sea of Japan and Oyashio Current**														
Iwaya, Fukuoka	JPN	33°55′58″N, 130°41′08″E	2015	15 (Mg)	287	0.0	0.0	5.2	—	—	1.8	—	24	*S*. *Hashimoto*, *YK*, *MEF*
Ayaragi, Yamaguchi	JPN	34°0′37″N, 130°54′57″E	2015	0	16	—	—	0.0	—	—	—	—	—	*S*. *Hashimoto*, *YK*, *MEF*
Ube harbour, Yamaguchi	JPN	33°56′33″N, 131°14′44″E	2015	0	150	—	—	0.0	—	—	—	—	—	*S*. *Hashimoto*, *YK*, *MEF*
Ondo-no-Seto, Hiroshima	JPN	34°11′35″N, 132°32′05″E	2015	44 (Mg)	305	0.0	2.3	10.1	—	1.0	1.4	—	33	*YK*, *MEF*
Kamo River bridge, Takehara, Hiroshima	JPN	34°19′41″N, 132°53′52″E	2015	0	175	—	—	10.3	—	—	2.4	—	29	*H*. *Uchiumi*, *YK*, *SO*, *MEF*
Mukaishima, Hiroshima	JPN	34°22′59″N, 133°10′22″E	2015	0	26	—	—	0.0	—	—	—	—	—	*M*. *Urata*, MEF
Niihama, Ehime	JPN	33°58′56″N, 133°17′37″E	2015	0	265	—	—	10.6	—	—	1.8	—	30	*S*. *Hashimoto*, *YK*, *MEF*
Tarumi Bay, Kagawa	JPN	34°22′41″N, 133°56′42″E	2015	0	300	—	—	6.3	—	—	1.3	—	21	*S*. *Hashimoto*, *YK*, *MEF*
Hiwasa, Tokushima (location 1)	JPN	33°43′47″N, 134°31′59″E	2015	0	100	—	—	0.0	—	—	—	—	—	*S*. *Hashimoto*, *YK*, *MEF*
Hiwasa, Tokushima (location 2)	JPN	33°44′33″N, 134°33′23″E	2015	0	115	—	—	0.8	—	—	1	—	1	*S*. *Hashimoto*, *YK*, *MEF*
Ofunato, Iwate	JPN	39°03′19″N, 141°43′27″E	2015	0	125	—	—	1.6	—	—	1	—	1	*YK*, *MEF*
Onizawa, Iwate	JPN	39°05′14″N, 141°48′42″E	2015	0	76	—	—	1.3	—	—	1	—	—	*YK*, *MEF*
Sakihama, Iwate	JPN	39°05′56″N, 141°51′36″E	2015	0	100	—	—	1.0	—	—	1	—	—	*YK*, *MEF*
		**Region total**		**59 (Mg)**	**2040**							**0**	**139**	
**Pacific coast of North America**														
Belfair State Park, Hood Channel	USA	47°25′45″N, 122°52′24″W	2015	11 (Mt)	55	0.0	0.0	10.9	—	—	5.8	—	22	*KMW*, *LNSS*
Harstine Island	USA	47°14′58″N, 122°52′14″W	2015	20 (Mt)	20	0.0	0.0	5.0	—	—	1.0	—	1	*KMW*, *LNSS*
Grappler Inlet, Bamfield	CAN	48°49′45″N, 125°7′40″W	2015	23 (Mt)	0	0.0	4.3	—	—	1.0	—	—	0	*KMW*, *LNSS*
Piper’s Lagoon, Nanaimo	CAN	49°13′34″N, 123°56′59″W	2015	20 (Mt)	64	0.0	25.0	29.7	—	1.6	1.6	—	5	*KMW*, *LNSS*
Fanny Bay	CAN	49°30′23″N, 124°49′35″W	2015	20 (Mt)	0	0.0	20.0	—	—	2.3	—	—	2	*KMW*, *LNSS*
Morning Beach, Denman Island	CAN	49°35′56″N, 124°49′31″W	2015	0	49	—	—	14.3	—	—	2.3	—	0	*KMW*, *LNSS*
Manson’s Landing, Cortes	CAN	50°04′17″N, 124°58′50″W	2015	10 (Mt)	30	0.0	60	6.7	—	6.0	1.0	—	8	*KMW*, *LNSS*
		**Region total**		**104 (Mt)**	**218**							**0**	**38**	
**Mediterranean Sea and Adriatic Sea**														
Bouzigues	FRA	43°26′51″N, 3°39′06″E	2016	110 (Mg)	58	20.0	0.0	1.7	1.3	—	2.0	30	—	*PCL*, *MEF*
Balaruc le Vieux	FRA	43°27′36″N, 3°40′50″E	2016	0	10	—	—	0.0	—	—	—	—	—	*PCL*, *MEF*
Balaruc les Bains	FRA	43°26′23″N, 3°40′59″E	2016	49 (Mg)	10	16.3	2.0	0.0	1.0	1.0	—	1	—	*PCL*, *MEF*
Port des Heures Claires, Istres	FRA	43°29′51″N, 4°59′56″E	2016	235 (Mg)	0	11.9	0.0	—	1.1	—	—	30	—	*PCL*, *MEF*
Plage de Rouet	FRA	43°20′00″N, 5°10′22“E	2016	50 (Mg)	0	68.0	0.0	—	1.9	—	—	29	—	*PCL*, *MEF*
Cassis harbour	FRA	43°12′53″N, 5°32′14″E	2016	50 (Mg)	0	82.0	0.0	—	3.0	—	—	30	—	*PCL*, *MEF*
Livorno harbour	ITA	43°32′45″N, 10°18′14″E	2016	50 (Mg)	0	60.0	0.0	—	2.8	—	—	30	—	*PCL*, *OB*
Piombino	ITA	42°57′19″N, 10°35′51″E	2016	100 (Mg)	0	0.0	0.0	—	—	—	—	—	—	*PCL*, *OB*
Lido di Ostia harbour	ITA	41°44′17″N, 12°14′54″E	2016	100 (Mg)	0	0.0	0.0	—	—	—	—	—	—	*PCL*, *OB*
Via del Pescatori, Ostia	ITA	41°43′08″N, 12°18′11″E	2016	100 (Mg)	0	0.0	0.0	—	—	—	—	—	—	*PCL*, *OB*
Trieste (subtidal)	ITA	45.76N, 13.58 or 13.59E	2014	238 (Mg)	0	0	0.0	—	—	—	—	—	—	*C*. *Manfrin*, MEF
Malinska	CRO	45°07′14″N, 14°31′13″E	2016	30 (Mg)	0	76.7	0.0	—	2.7	—	—	30	—	*E*. *van Veenendaal*, *OB*
Marjan peninsula	CRO	43°30′54″N, 16°24′18″E	2016	67 (Mg)	0	44.8	0.0	—	1.9	—	—	29	—	*E*. *van Veenendaal*, *OB*
Mitikas	GRE	39°00′08″N, 20°42′23″E	2016	150 (Mg)	0	0.0	0.0	—	—	—	—	—	—	*O*. *Müller*, *MAG*
Preveza	GRE	38°57′36″N, 20°45′29″E	2016	150 (Mg)	0	0.0	0.0	—	—	—	—	—	—	*O*. *Müller*, *MAG*
Aliakmonas National Park	GRE	40°33′07″N, 22°44′29″E	2016	150 (Mg)	0	0.0	0.0	—	—	—	—	—	—	*O*. *Müller*, *MAG*
Angelochori	GRE	40°29′44″N, 22°48′56″E	2016	150 (Mg)	0	0.0	0.0	—	—	—	—	—	—	*O*. *Müller*, *MAG*
Nea Kallikratia	GRE	40°18′37″N, 23°03′30″E	2016	150 (Mg)	0	0.0	0.0	—	—	—	—	—	—	*O*. *Müller*, *MAG*
Neos Marmaras	GRE	40°05′36″N, 23°46′53″E	2016	150 (Mg)	0	0.0	0.0	—	—	—	—	—	—	*O*. *Müller*, *MAG*
Rhodes	GRE	36°16′21″N, 27°49′29″E	2016	90 (Bp)	0	0.0	0.0	—	—	—	—	—	—	*PCL*
		**Region total**		**2079 (Mg)** **90 (Bp)**	**78**							**209**	**0**	
**Atlantic coast of Europe**														
Tavira	PRT	37°07′02″N, 7°37′46″W	2014	289 (Mg)	0	1.4	5.6	—	1.0	1.2	—	—	31	*F*. *Batista*, *MEF*
Carreço	PRT	41°44′0″N, 8°52′23″W	2014	231 (Mg)	0	0	0.4	—	—	1.0	—	—	—	*C*. *de la Vega*, MEF
Biarritz	FRA	43°31′55″N, 1°31′44″W	2016	76 (Me)	0	1.3	0.0	—	1.0	—	—	—	—	*O*. *Müller*, *MAG*
Hossegor	FRA	43°39′20″N, 1°26′19″W	2016	100 (Me)	0	0.0	0.0	—	—	—	—	—	—	*O*. *Müller*, *MAG*
Vieux Boucau	FRA	43°47′07″N, 1°24′59″W	2016	101 (Me)	0	0.0	0.0	—	—	—	—	—	—	*O*. *Müller*, *MAG*
Arcachon Bay	FRA	44°43′18″N, 1°11′19″E	2014	264 (Me)	452	NA	NA	4.9	NA	NA	1.6	—	31	*X*. *de Montaudouin*
Ronce les Bains, La Tremblade	FRA	45°48′00″N, 1°10′00″W	2015	NA (Me)	0	90–95	0.0	—	3.87	—	—	22	—	*IA*
Ré island, La Rochelle	FRA	46°09′41″N, 1°21′52″W	2013	54 (Me)	0	74.1	9.3	—	2.2	1.0	—	16	1	*M*. *Paar*, MEF
Audierne Bay	FRA	47°57′13″N, 4°24′54″W	2015	20 (Me)	0	0.0	0.0	—	—	—	—	—	—	*IA*
		**Region total**		**520 (Mg)** **> 615 (Me)**	**452**							**38**	**63**	
**Wadden Sea and North Sea**														
Balgzand	NLD	52°55′50″N, 4°54′09″E	2012	80 (Me)	80	8.8	58.8	33.8	1.0	2.8	4.5	5	29	*R*. *Nauta*, *MAG*
De Cocksdorp, Texel	NLD	53°08′51″N, 4°54′11″E	2012	80 (Me)	80	12.5	46.3	25.0	1.0	1.9	3.9	7	30	*R*. *Nauta*, *MAG*
Ameland	NLD	53°25′54″N, 5°43′22″E	2012	80 (Me)	80	53.8	62.5	16.3	1.6	4.1	7.0	30	30	*R*. *Nauta*, *MAG*
Schiermonnikoog	NLD	53°28′02″N, 6°12′34″E	2012	80 (Me)	80	27.5	73.8	17.5	1.3	4.8	9.8	16	30	*R*. *Nauta*, *MAG*
Horumersiel	DEU	53°41′40″N, 8°01′55″E	2012	80 (Me)	80	22.5	41.3	21.3	1.3	1.9	9.2	21	32	*R*. *Nauta*, *MAG*
Norddeich	DEU	53°40′57″N, 7°16′24″E	2012	80 (Me)	80	66.3	62.5	21.3	2.6	3.2	10.6	45	28	*R*. *Nauta*, *MAG*
Puan Klent, Sylt	DEU	54°47′54″N, 8°18′11″E	2012	80 (Me)	80	93.8	60	17.5	4.0	2.6	4.6	30	25	*R*. *Nauta*, *MAG*
Königshafen, Sylt	DEU	55°01′45″N, 8°26′03″E	2012	80 (Me)	80	78.8	1.3	0.0	3.2	1.0	—	23	—	*R*. *Nauta*, *MAG*
Helgoland (subtidal)	DEU	54°10′31″N, 7°53′41″E	2014	150 (Me)	0	0.0	0.0	—	—	—	—	—	—	*AWI divers*, MEF
Helgoland	DEU	54°10′45″N, 7°53′22″E	2014	0	20	—	—	0.0	—	—	—	—	—	*MEF*
		**Region total**		**790 (Me)**	**660**							**177**	**204**	
**Baltic Sea**														
Kiel Bülk	DEU	54°27′15″N, 10°11′53″E	2014	75 (Me)	0	0.0	0.0	—	—	—	—	—	—	*A*. *Pansch*, MEF
Kiel Fjord	DEU	54°19′49″N, 10°08′59″E	2014	115 (Me)	0	0.0	0.0	—	—	—	—	—	—	*A*. *Pansch*, MEF
		**Region total**		**190 (Me)**	**0**							**0**	**0**	
		**TOTAL ALL LOCATIONS**		**2658 (Mg)** **> 1595 (Me)** **104 (Mt)** **90 (Bp)**	**5604**							**424**	**444**	

All hosts were collected in harbors or on natural mussel or oyster beds, which were almost all situated in intertidal areas (unless indicated). N_host_: total number of hosts dissected; N_parasites_: number of parasites successfully sequenced for cytochrome-*c*-oxidase 1; Collectors and dissectors: collectors are indicated in italics, dissectors by underlining. Note that no *M*. *intestinalis* was found in Pacific oysters in this study, therefore the column representing this host-parasite combination was omitted from the table.

### Invasion genetics of *Mytilicola* spp

**Figure 2:**
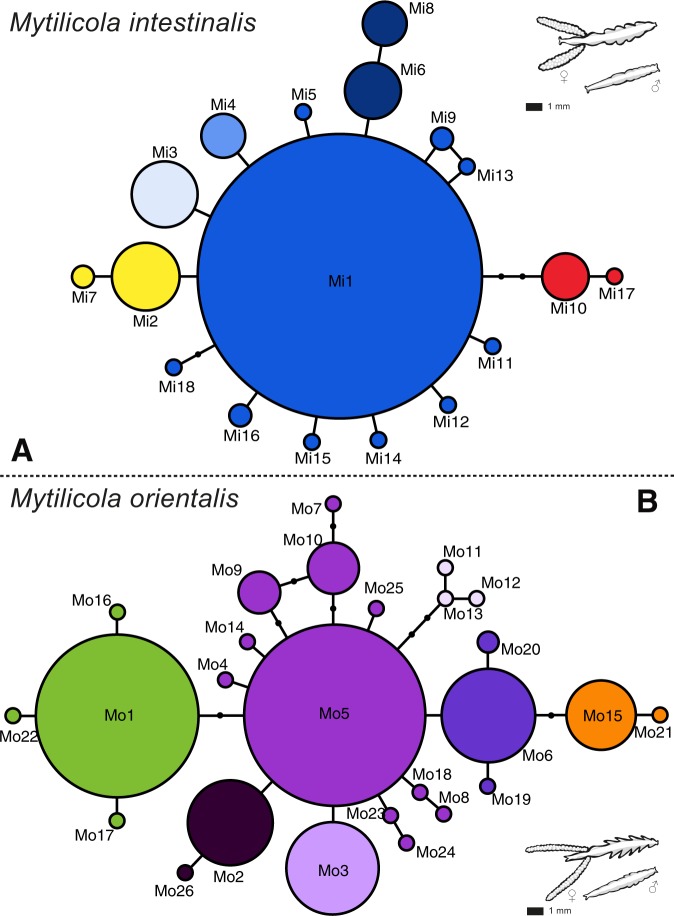
Minimum spanning networks for COI haplotypes (circles) in (**A**) *Mytilicola intestinalis* and (**B**) *M*. *orientalis*. Branch length is proportional to the number of nucleotide differences (the shortest branches represent one nucleotide difference). The black dots on the branches represent missing haplotypes. Frequency of observation is proportional to circle area. Colors represent large scale differentiation between clusters of haplotypes and correspond with the pie charts in Fig. 4. Figure 2 was drawn in Adobe Illustrator based on the Arlequin output list of OTU differences.

**Figure 3:**
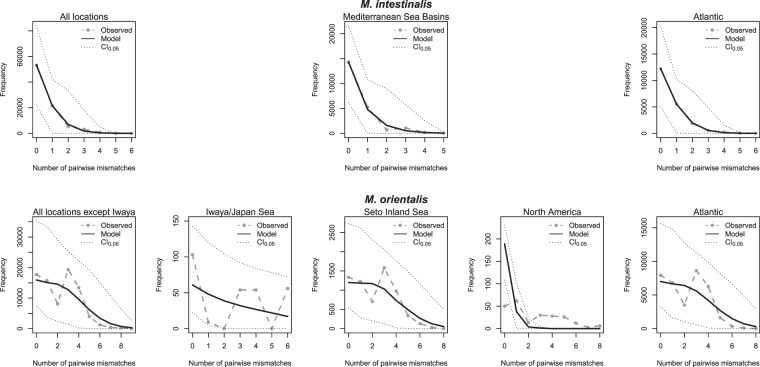
Mismatch distributions of *Mytilicola intestinalis* (top row) and *Mytilicola orientalis* (lower row). From left to right: mismatch distributions based on population differences (*M*. *intestinalis*: all locations pooled; *M*. *orientalis*: Iwaya separate from the rest of the locations), mismatch distributions in the putative native region and in the invaded region(s). The sharp decline in *M*. *intestinalis* is indicative of population expansion after a bottleneck or selective sweep; the bimodal distributions in *M*. *orientalis* are indicative of stable demographics.

**Figure 4:**
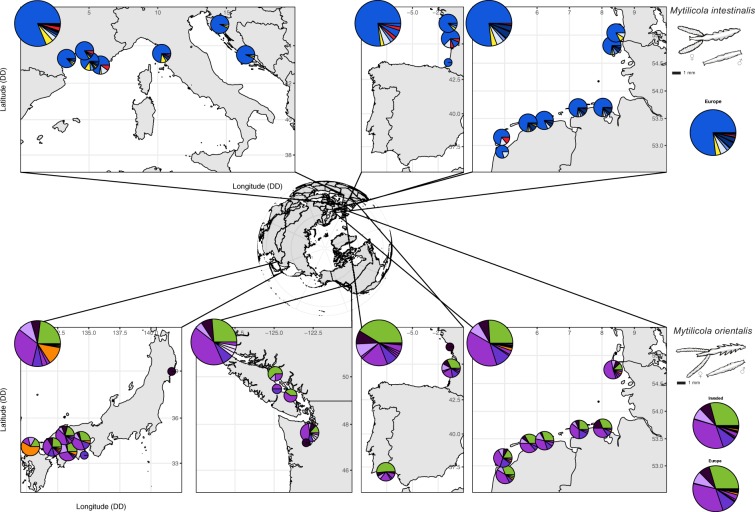
Maps showing pie charts of cytochrome-*c*-oxidase 1 (COI) haplotype frequencies in the invasive parasite species *Mytilicola intestinalis* and *Mytilicola orientalis*. Shown plotted overlaying the maps are pie charts for the different sampling locations, while the large pie charts in the corner of the maps represent the grouped frequencies for all locations in that map. On the side, the large pie charts represent the grouped frequencies for all invaded European locations for *M*. *intestinalis* and *M*. *orientalis* (“Europe”), and for all invaded locations for *M*. *orientalis* that includes the North American samples (“Invaded”). Note that the pie charts from the Mediterranean and Japan represent the overall frequencies of all sampled native sampling locations for *M*. *intestinalis* and *M*. *orientalis*, respectively. Pie charts are smaller for sampling locations with fewer than 15 individuals, which were not included in our analyses. Colors correspond to large scale differentiation between clusters of haplotypes in the minimum spanning networks presented in Fig. 2. Pie charts were made in R statistical environment version 3.4.3^83^, maps were plotted with R packages rworldmap^67^ and ggplot^68^ and Fig. 4 was assembled in Adobe Illustrator.

A total of 424 *M*. *intestinalis* and 444 *M*. *orientalis* were successfully sequenced, which resulted in the detection of 18 and 26 haplotypes, respectively (Tables S1 and S2) (Genbank accession No. MN334483-MN334526). The minimum spanning network of *M*. *intestinalis* had a star-like structure with one central, highly abundant haplotype from which several haplotypes were separated by a few mutational steps (Fig. 2A). The star-like structure of the network suggests recent demographic expansion, as is also suggested by the negative Tajima’s D and Fu’s F_S_ values which were significant for most locations (Table 2), and by the mismatch distributions (Fig. 3). Nine of the ten non-singleton haplotypes were shared between the putative native and invaded range (Figs 2A, 4 and Supplementary Table S1); only haplotype Mi16 was solely found in the invaded range (La Rochelle, Supplementary Table S1).

**Table 2:** Tajima’s D and Fu’s F_s_ neutrality tests for partial cytochrome-*c*-oxidase 1 sequences in *Mytilicola intestinalis* and *M*. *orientalis*, based on 10,000 simulations.

Locations	Tajima’s D	Fu’s F_S_	Locations	Tajima’s D	Fu’s F_S_
***M***. ***intestinalis***, **putative native region**			***M***. ***orientalis***, **putative native region**		
Marjan, Croatia	−1.509*	−2.312**	Iwaya, Japan	1.24	3.06
Malinska, Croatia	−1.507*	−2.355*	Ondo, Japan	−0.482	−1.11
Livorno, Italy	−1.871*	−3.027**	Takehara, Japan	−1.12	−2.23
Cassis, France	−0.8597	0.0501	Niihama, Japan	−0.372	−0.109
Istres, France	−1.749**	−2.054	Tarumi, Japan	−0.220	−0.0780
Rouet, France	−1.662*	−5.145***			
Bouzigues, France	−1.889**	−4.411***			
***M***. ***intestinalis***, **invaded region**			***M***. ***orientalis***, **invaded region**		
La Rochelle, France	−0.9778	−0.7836	Belfair, USA	−1.12	−2.22
La Tremblade, France	−1.878**	−2.206*	Tavira, Portugal	0.125	0.110
Ameland, Netherlands	−1.658*	−4.526**	Arcachon, France	−0.878	−1.15
Schiermonnikoog, Netherlands	−1.349	−1.867*	Balgzand, Netherlands	−0.230	−0.319
Norddeich, Germany	−1.638*	−3.161*	De Cocksdorp, Netherlands	−1.04	−1.50
Horumersiel, Germany	−1.159	−1.259*	Ameland, Netherlands	0.0919	0.518
Puan Klent, Germany	−1.758*	−4.899***	Schiermonnikoog, Netherlands	−0.208	−0.266
Königshafen, Germany	−0.864	−0.8722	Norddeich, Germany	−0.420	−0.558
			Horumersiel, Germany	0.178	0.655
			Puan Klent, Germany	−1.538*	−3.22*

Level of significance is indicated (*p* < 0.05*, *p* < 0.01** and *p* < 0.001***). Only samples with more than 15 individuals were included in the analyses; see column N_parasites_ in Table 1.

In contrast, the minimum spanning network of *M*. *orientalis* was relatively complex with two common frequent haplotypes that both showed associated derived haplotypes (Fig. 2B). With the exception of one location in Japan, all locations had one of the main haplotypes as its most frequent haplotype. A small group of related singleton haplotypes was found only in North America (light purple colored haplotypes Mo11-Mo13, Figs 2B, 3 and Supplementary Table S2). This type of network is more typically associated with stable demographics, also suggested by the non-significant Tajima’s D and Fu’s F_S_ values (Table 2), and by the mismatch distributions (Fig. 3). Only one population (Puan Klent, Germany), representing the northern invasion front of *M*. *orientalis* at time of collection, stood out with significant negative Tajima’s D and Fu’s F_S_ values (Table 2).

Because of the long geological history of the Mediterranean Sea Basins, its different hydrographic regimes and the short dispersive larval phase of *M*. *intestinalis*, we expected a high genetic diversity of *M*. *intestinalis* in this region and population genetic structure between the different basins. The overall level of population differentiation for *M*. *intestinalis* was estimated at Φ_ST_ = 0.01468 (*p* = 0.0108) and *F*_ST_ = 0.00848 (n.s.), with an overall population differentiation in the Mediterranean Sea Basins of Φ_ST_ = 0.01531 (*p* = 0.0323) and *F*_ST_ = 0.02397 (*p* = 0.0325), and in the invaded region of Φ_ST_ = 0.01081 (n.s.) and *F*_ST_ = −0.00544 (n.s.) (Table 3).

**Table 3:** Results from analyses of molecular variance (AMOVA) on partial cytochrome-*c*-oxidase 1 (COI) sequences in *Mytilicola intestinalis* and *M*. *orientalis* with mitochondrial fixation index Φ_ST_ and with conventional *F*-statistics.

Locations		df	SS	VC	Φ_ST_/*F*_ST_	*p*-value
***Mytilicola intestinalis***, **Φ**_**ST**_						
All	among	14	5.318	0.00402		
	within	396	106.955	0.27009		
	total	410	112.273	0.27411	0.01468	0.01075
Mediterranean	among	6	2.147	0.00381		
	within	201	49.199	0.24477		
	total	207	51.346	0.24858	0.01531	0.03226
Introduced	among	7	2.638	0.00324		
	within	195	57.756	0.29618		
	total	202	60.394	0.29942	0.01081	0.10264
***Mytilicola intestinalis***, ***F***_**ST**_						
All	among	14	3.167	0.00157		
	within	396	72.622	0.18339		
	total	410	75.622	0.18496	0.00848	0.13436
Mediterranean	among	6	1.707	0.00404		
	within	201	33.062	0.16449		
	total	207	34.062	0.16853	0.02397	0.03248
Introduced	among	7	1.228	−0.00110		
	within	195	39.560	0.20287		
	total	202	40.788	0.20177	−0.00544	0.63683
***Mytilicola orientalis***, **Φ**_**ST**_						
All	among	14	39.374	0.06064		
	within	410	449.440	1.09620		
	total	424	488.814	1.15683	0.05242	<0.00001
Japan	among	4	18.935	0.13168		
	within	132	151.576	114830		
	total	136	170.511	1.27998	0.10287	<0.00001
Introduced	among	9	16.007	0.02458		
	within	278	297.865	1.07146		
	total	287	313.872	1.09604	0.02243	0.03030
***Mytilicola orientalis***, ***F***_**ST**_						
All	among	14	10.084	0.01188		
	within	410	157.436	0.38399		
	total	424	167.520	0.39587	0.03002	<0.00001
Japan	among	4	4.672	0.02866		
	within	132	50.459	0.38227		
	total	136	55.131	0.41112	0.07019	<0.00001
Introduced	among	9	4.475	0.00391		
	within	278	106.977	0.38481		
	total	287	111.451	0.38871	0.01005	0.11604

Only samples with more than 15 individuals were included. Abbreviations: df = degrees of freedom; SS = sum of squares; VC = variance component, Φ_ST_ and *F*_ST_ = overall fixation index.

For *M*. *orientalis*, we adopted the default expectation of high genetic diversity in the whole native region and genetic differentiation among populations in the absence of detailed knowledge of the distribution in the native range. Because of the many transport occurrences of Pacific oysters to North America and Europe, we did not expect to find a reduction in *M*. *orientalis* genetic diversity in the invaded regions. The overall level of population differentiation for *M*. *orientalis* was higher and estimated at Φ_ST_ = 0.05242 (*p* < 0.00001) and *F*_ST_ = 0.03002 (*p* < 0.00001), with an overall population differentiation in Japan of Φ_ST_ = 0.10287 (*p* < 0.00001) and *F*_ST_ = 0.07019 (*p* < 0.00001), and in the introduced region of Φ_ST_ = 0.02243 (*p* = 0.0303) and *F*_ST_ = 0.01005 (n.s.) (Table 3).

The small, overall genetic differentiation in *M*. *intestinalis* could not be attributed to any single location or group of locations based on the pairwise population comparisons because none of the comparisons were significant after Bonferroni correction (Supplementary Tables S3, S4). The hierarchical AMOVAs did not clarify this further, as they did not detect significant groupings on any of the hierarchical levels. Including Cassis into the Western Mediterranean group led to negative Φ_CT_ values (Table 4). When grouping Cassis into the Ligurian Sea, the percentage of variation among groups was 0.38%, among populations within groups 1.24%, and within populations 98.38%, resulting in Φ_CT_ = 0.0038 (n.s.), Φ_SC_ = 0.01247 (n.s.) and Φ_ST_ = 0.0162 (*p* = 0.0480) (Table 4). Using classical *F*-statistics, the percentage of variation among groups was greater at 2.07%, smaller among populations within groups at 0.84%, and similar within populations at 97.12%, resulting in *F*_CT_ = 0.02069 (n.s.), *F*_SC_ = 0.00825 (n.s.) and *F*_ST_ = 0.02878 (n.s.) (Table 4). In both hierarchical AMOVAs using mitochondrial statistic Φ and conventional *F*-statistic, there was no significant difference between the Gulf of Lion, the Ligurian Sea and the Adriatic Sea. Therefore, the small amount of population structure that was detected in the overall test cannot clearly be attributed to one of the Mediterranean Sea Basins, nor to one or a few individual locations.

**Table 4:** Results from hierarchical analyses of molecular variance (AMOVA) based on pairwise differences, on partial cytochrome-*c*-oxidase 1 (COI) sequences in Mediterranean *Mytilicola intestinalis* with mitochondrial Φ-statistic and conventional *F*-statistic.

	df	SS	VC	% var	*p*-value	Φ or *F*-statistic
***AMOVA 1: Gulf of Lion (Bouzigues***, ***Istres***, ***Rouet***, ***Cassis) vs***. ***Ligurian Sea (Livorno) vs***. ***Adriatic Sea (Marjan***, ***Malinska)***						
among groups	2	0.476	−0.00303	−1.22	0.76653	Φ_CT_ = −0.01224
among populations within groups	4	1.671	0.00583	2.35	0.04089	Φ_SC_ = 0.02325
within populations	201	49.199	0.24477	98.87	0.04465	Φ_ST_ = 0.01130
total	207	51.346	0.24757			
***AMOVA 1: Gulf of Lion (Bouzigues***, ***Istres***, ***Rouet***, ***Cassis) vs***. ***Ligurian Sea (Livorno) vs***. ***Adriatic Sea (Marjan***, ***Malinska)***						
among groups	2	0.776	0.00261	1.54	0.20634	*F*_CT_ = 0.01543
among populations within groups	4	0.931	0.00230	1.36	0.13525	*F*_SC_ = 0.01378
within populations	201	33.062	0.16449	97.10	0.03040	*F*_ST_ = 0.02899
total	207	34.769	0.16940			
***AMOVA 2: Gulf of Lion (Bouzigues***, ***Istres***, ***Rouet) vs***. ***Ligurian Sea (Cassis***, ***Livorno) vs***. ***Adriatic Sea (Marjan***, ***Malinska)***						
among groups	2	0.801	0.00094	0.38	0.33257	Φ_CT_ = 0.00378
among populations within groups	4	1.346	0.00309	1.24	0.14168	Φ_SC_ = 0.01247
within populations	201	49.199	0.24477	98.38	0.04802	Φ_ST_ = 0.011620
total	207	51.346	0.24880			
***AMOVA 2: Gulf of Lion (Bouzigues***, ***Istres***, ***Rouet) vs***. ***Ligurian Sea (Cassis***, ***Livorno) vs***. ***Adriatic Sea (Marjan***, ***Malinska)***						
among groups	2	0.887	0.00350	2.07	0.16406	*F*_CT_ = 0.02069
among populations within groups	4	0.821	0.00137	0.84	0.23287	*F*_SC_ = 0.00825
within populations	201	33.062	0.16449	97.12	0.03168	*F*_ST_ = 0.02878
total	207	34.769	0.16936			

Only samples with more than 15 individuals were included; see column N_parasites_ in Table 1. Abbreviations: df = degrees of freedom; SS = sum of squares; VC = variance component, % var = percentage of variation.

In contrast to *M*. *intestinalis*, there was strong and significant genetic differentiation between locations in the native region of *M*. *orientalis*. Iwaya, located in the Sea of Japan, was strongly differentiated from nearly all other sampling locations along the Seto Inland Sea (Φ_ST_ = 0.215–0.290, Supplementary Table S5; *F*_ST_ = 0.165–0.216, Supplementary Fig. S3, Supplementary Table S6). Only the pairwise population comparison between Iwaya (Fukuoka Prefecture) and Tarumi (Kagawa Prefecture) was not significant after Bonferroni correction (Φ_ST_ = 0.220, *p* = 0.00129 > Bonferroni corrected *p*-level of 0.000476, Supplementary Table S5). However, with conventional *F*-statistics, also this pairwise comparison was significantly different (*F*_ST_ = 0.191, *p* = 0.00040 < Bonferroni corrected *p*-level of 0.000476, Supplementary Table S6). No genetic differentiation was detected between the locations sampled along the Seto Inland Sea, i.e., Takahara, Ondo, Niihama and Tarumi. All pairwise population comparisons between the Sea of Japan (Iwaya) and locations from the invaded regions in North America and Europe were significant after Bonferroni correction and showed strong genetic differentiation (Φ_ST_ = 0.228–0.344, Supplementary Table S5; *F*_ST_ = 0.187–0.247, Supplementary Table S6). No significant pairwise population differences were detected between locations in the invaded regions.

**Figure 5:**
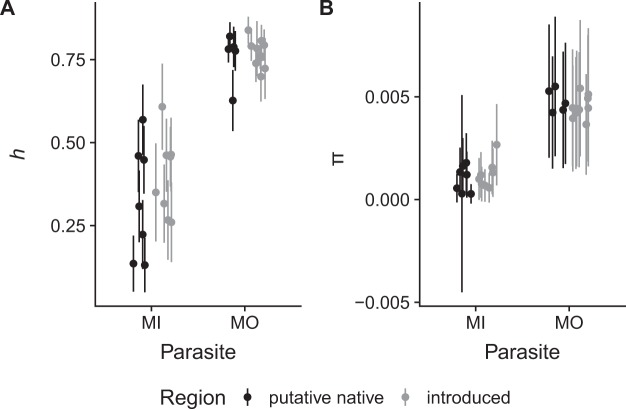
Haplotype diversity *h* and nucleotide diversity *π* of *Mytilicola intestinalis* (MI) and *Mytilicola orientalis* (MO). Putative native ranges are indicted in grey and invaded ranges in black. Bars represent standard deviations. Only samples with more than 15 individuals were included. See Supplementary Tables S1 and S2 for *π* and *h* values of individual locations underlying this figure.

We did not detect significant differences in haplotype diversity (*h*) between the putative native regions and the introduced regions (ANOVA, F_1, 26_ = 1.1081, *p* = 0.302) (Fig. 5A). Haplotype diversity for *M*. *intestinalis* ranged from 0.131 to 0.569 in the putative native region and from 0.260 to 0.608 in the invaded region (Supplementary Table S1), while for *M*. *orientalis*, *h* ranged from 0.627 to 0.820 in its putative native region, Japan, (all locations with >15 individuals) and from 0.699 to 0.839 in its introduced region (Supplementary Table S2). Similarly, no significant differences were detected for nucleotide diversity (*π*) between the putative native region and the invaded regions (ANOVA, F_1,26_ = 0.0284, *p* = 0.867). Nucleotide diversity for *M*. *intestinalis* ranged from 0.000276 to 0.00179 in the putative native region, and from 0.000572 to 0.00267 in the introduced region (Fig. 5B, Supplementary Table S1), and *M*. *orientalis’* nucleotide diversity ranged from 0.00423 to 0.00550 in Japan and from 0.00366 to 0.00541 in the introduced regions (Fig. 5B, Supplementary Table S2). However, both haplotype diversity and nucleotide diversity were significantly larger for *M*. *orientalis* than for *M*. *intestinalis* (ANOVA *h*: F_1, 26_ = 99.2662, *p* < 0.00001; ANOVA *π*: F_1, 26_ = 251.7502, *p* < 0.00001). The interaction term in the linear models (species × region) was not significant for haplotype diversity *h* or nucleotide diversity *π* (ANOVA *h*: F_1, 26_ = 0.5634, *p* = 0.460; ANOVA *π*: F_1, 26_ = 1.3178, *p* = 0.261), indicating that genetic diversity differed consistently between species.

## Discussion

Our study utilized the consecutive invasions of two congeneric invasive parasites to align the invasion history derived from their reported spread in the literature with their phylogeography based on molecular markers. Based on mitochondrial haplotype diversity, we found that the native range and the invasion route of *Mytilicola orientalis* match the classical pattern of a recent invasion described in the literature, while the phylogeography of *Mytilicola intestinalis* did not match our literature-based expectations due to low mtDNA genetic diversity. While several scenarios could explain the low genetic diversity, we cannot identify the origin of the species, which potentially also makes it a cryptogenic species in the Mediterranean Sea Basins.

### *M*. *orientalis* phylogeography supports invasion history

Our study confirmed the coastal seas of Japan as the native region of *M*. *orientalis*, because of the significant population genetic structure between the Seto Inland Sea and Iwaya in the Sea of Japan, and the stronger overall population differentiation in Japan than in the invaded regions. This was expected based on the invasion history derived from our literature survey. The invasion of *M*. *orientalis* was extremely long-distance, crossing both the Pacific and Atlantic Ocean via aquaculture transports, followed by a relatively rapid spread through the invaded regions (Fig. 1B, Resource 2) that is still ongoing^34^ (Feis, unpubl. data).

Pacific oysters have been imported from Japan to locations across the globe, with Miyagi Prefecture as the main overseas export area and Kumamota and Hiroshima Prefectures including the Seto Inland Sea^35^ contributing smaller amounts^35^. Oyster seed is regularly transported between the regions largely homogenizing the genetic diversity and restricted gene flow has only been reported for the Nagasaki and Kumano regions^36^. *Mytilicola orientalis* hitchhiked with oyster transports to North America^16^ and to Europe^15,37,38^, and our population genetic analyses support these patterns in the native range and invaded regions: the North American and European samples could not be distinguished from the Seto Inland Sea samples from Japan, while the sample from Iwaya in the Sea of Japan was significantly different.

Within the invaded region in Europe, *M*. *orientalis* showed more resemblance in the MDS plots between North America and the northern Wadden Sea invasion: Belfair (representing North America) and Puan Klent (representing the northern Wadden Sea oyster invasion). This pattern represents a small mismatch with the phylogeography of the host^19,20^ and might suggest that other vectors than oyster transports contributed to *M*. *orientalis’* secondary spread in the Wadden Sea. For example, the wider host range of *M*. *orientalis* and spillover to native bivalves^18^ could have further sped up and modified its spread. In the Mediterranean, the distribution of *M*. *orientalis* is disjunctive, with presence at the Thau lagoon in France since 1979^39^ and in the northern Adriatic^40^, but an absence in between (Table 1). This patchy distribution pattern probably reflects the long-distance transports of oysters and *M*. *orientalis’* ongoing invasion, and furthermore demonstrates that *Mytilicola* spp. do not colonize rapidly over large distances unaided by anthropogenic factors.

In conclusion, *M*. *orientalis* was likely co-introduced in large numbers or via multiple introductions, based on the equally high genetic diversity in native and invaded regions. Thus, the invasion history from the native region along the reported invasion route of *M*. *orientalis* from our literature survey matches well with the resulting phylogeography based on COI haplotypes.

### No differentiation in the putative native range of *M*. *intestinalis*

Based on our literature survey, the invasion of Atlantic coasts of Europe by *M*. *intestinalis* can be characterized by local introductions at seaports and harbor areas, and a slow natural spread afterwards. While *M*. *intestinalis* was thought to originate from the Mediterranean Sea Basins, our genetic data based on mtDNA could not confirm this as a source of the invasion. Even though the overall level of population genetic structure in the Mediterranean was significant, it was very low. Zooming in on this small difference, however, we did not detect a significant difference among Mediterranean Sea Basins based on hierarchical AMOVAs nor between any pairwise comparison between Mediterranean samples. The absence of clear population genetic differentiation in the Mediterranean is striking. If the Mediterranean Sea Basins were the native distribution range of *M*. *intestinalis*, the long geological history of the basins should have resulted in significant population structure. Although no uniform phylogeographic pattern in other marine taxa in the Mediterranean Sea Basins has been found^41^, for many species, the topography and oceanographic conditions of the Strait of Otranto and the Siculo-Tunisian Strait represent a break that typically causes population genetic differentiation between the Adriatic, Ionian and Ligurian Seas (e.g.^42–44^). Likewise, genetic breaks were found near Marseille, separating the Ligurian Sea and Gulf of Lion, especially for species with lower dispersal ability^44–46^. As the short-lived pelagic larvae of *Mytilicola* limit the potential for natural dispersal^12,23^, population differentiation among the long-established Mediterranean populations should have been maintained. Especially, since breaks in the distribution of mussel hosts introduce additional barriers for *M*. *intestinalis* larval dispersal. This limited dispersive ability of *M*. *intestinalis* can also be observed in the the literature-based invasion data (Resource 1), showing a slow spread upon invasion in the Dutch Wadden Sea and Scottish lochs.

### Low mtDNA variability masks the origins of *M*. *intestinalis*

There are several possible explanations for the observed absence of population genetic differentiation between the Mediterranean *M*. *intestinalis* samples and the overall low genetic diversity compared to *M*. *orientalis*.

A bottleneck in *M*. *intestinalis* may have occurred prior to its invasion, which would have reduced standing genetic variation^47–50^. Such a scenario is supported by the star-like haplotype network, relatively low genetic diversity and significant negative Tajima’s D statistic that indicates recent population expansion or recovery from an ancient bottleneck after which diversity slowly recovered, creating a star-like haplotype network at the start. However, such a bottleneck must have happened fairly recent to prevent the build up of genetic differentiation over extended periods of time.

Alternatively, anthropogenic shuffling of native genetic diversity might explain the lack of population genetic structure in *M*. *intestinalis* within the Mediterranean. Like *M*. *orientalis*’ invasion, aquaculture transports might also be a cause here. However, the typically small size of translocated mussels (‘mussel spat’) contests this. The number of parasites is usually proportional to the shell length of mussels^51,52^ and very young mussels are rarely infected^12^. Moreover, Mediterranean commercial mussels are usually grown in hanging cultures, which are considerably less vulnerable to parasite infections than mussels grown on the benthos. These mussels are also replaced yearly by a new parent stock, which makes it difficult for *M*. *intestinalis* to build up a dense population (reviewed in^12^). Nevertheless, transportation of spat between Mediterranean regions does occur and could be contributing to or have contributed in the past to the homogenization of its parasite fauna. Other human activities related to mussel dispersal are ballast water and ship hull fouling^53^. Ship hull fouling was seen as the primary route by which *M*. *intestinalis* invaded the German and British coasts (e.g.^23^) and explained its disjunctive distribution there (reviewed in^12^). Higher resolution of more independent genetic markers might allow for detecting differentiation. Nevertheless, while anthropogenic shuffling could explain the lack of population genetic structure in the Mediterranean and Adriatic Seas, it cannot explain the overall low genetic diversity compared to *M*. *orientalis*.

Finally, *M*. *intestinalis* may be an introduced species in the Mediterranean Sea Basins, which would explain the lack of population genetic structure and the indications for population expansion. A few hints supporting this scenario can be found in literature. *Mytilicola intestinalis* was first described in 1902 and 1903 from two important ports for shipping, Trieste and Gravosa (both Adriatic Sea)^54,55^ – around 40 years after the opening of the Suez Canal (November 1869), an event that led to the invasion of many marine species into the Mediterranean (Lessepsian migration, e.g.^56,57^). In 1902, nearly all dissected mussels at those Adriatic sites were infected by *M*. *intestinalis*, with up to 50 parasites in one host^54,55^. Prevalences and intensities that high are typically seen in mussel populations that have recently invaded – e.g., maximum intensity of up to 14 parasites in Germany^10^, up to 59 in the UK^58^ and up to 70 in the Netherlands^59^. In 1953, *M*. *intestinalis* could not be found in the same locations where A. Steuer initially described the species^60^, and we also did not find *M*. *intestinalis* in > 200 mussels from Trieste (Table 1). Similarly, in Mare Piccolo, Taranto (Italy), *M*. *intestinalis* had been a sudden pest before 1932, but authorities successfully eradicated it^61^. At the same site, a prevalence of 6.7% and mean intensity 1.8 (n = 106) was found in 1953^62^ but in 1996, *M*. *intestinalis* was absent (n = 31)^63^. The cases of Trieste and Taranto both may be interpreted as outbreaks upon initial invasion followed by low infestation over longer time scales, resembling the typical boom-bust dynamics of biological invasions before populations enter the comparatively stable adjustment phase^64^. Moreover, Adriatic harbors that were rarely visited by large vessels had no or low *M*. *intestinalis* prevalences, while mussels at large ports were all heavily infested^65^.

Because our data may not be consistent with the idea that *M*. *intestinalis* is native in the Mediterranean Sea Basins, we suggest that *M*. *intestinalis* should be regarded as a cryptogenic species (i.e., a species whose origin cannot readily be determined with the available data, *sensu* Carlton^66^). Along European Atlantic and North Sea coasts, however, *M*. *intestinalis* is clearly an invasive species, as there are records of times when *M*. *intestinalis* was not present and later invaded. The absence of such data in the Mediterranean neither proves nor disproves presence or absence of *M*. *intestinalis* in the Mediterranean Sea Basins before its taxonomic description. The higher prevalence around shipping ports and aquaculture areas could indicate that *M*. *intestinalis* is invasive, or that its distribution has been influenced by humans also in the Mediterranean. Such a scenario would also imply that its native host species may not be *Mytilus galloprovincialis*. Solving the puzzle whether *M*. *intestinalis* is native or is an invader in the Mediterranean would need explorative, basic parasitological work in potential native regions such as the southern and eastern Mediterranean Sea Basins, the Red Sea and potentially further, in combination with a population genetic study.

Our study shows that despite the large body of literature existing on these two common marine parasites of commercially important bivalves, hypotheses derived from literature-based invasion histories reports do not necessarily match conclusions based on population genetic demography. This indicates that some basic understanding of the invasion processes is lacking, and highlights the usefulness of population genetic studies to test anecdotal evidence of invasion histories. The native region and invasion route of *M*. *orientalis* were validated with the sampling and choice of marker in the current study: the native region of *M*. *orientalis* is Japan, and the parasite hitchhiked with its host to North America and Europe. Whether *M*. *orientalis* was introduced to Europe via Canada, or directly from Japan, or both, and how *M*. *orientalis* spread throughout Europe remains to be answered with the use of higher resolution nuclear genetic markers. In contrast, the native region of *M*. *intestinalis* could not be validated, because of low genetic diversity and lacking population structure within and among the Mediterranean Sea basins, which may point to *M*. *intestinalis* being an invader in the Mediterranean Sea Basins (cryptogenic species). The invasion histories and demographics thus differ between the two *Mytilicola* species, and this may have profound impacts on their further invasion and evolutionary trajectories, as well as their competition in the invaded regions where they now co-occur.

## Materials and Methods

### Literature review

To describe the distribution of both invasive *Mytilicola* species through time in putative native and invaded regions, we searched the Web of Science/Web of Knowledge and Google Scholar with the keywords “*Mytilicola intestinalis*” and “*Mytilicola orientalis*”. Further publications, including grey literature, were found by checking the references of each relevant publication (i.e., “snowballing”). All publications were screened for recorded absence or presence of *Mytilicola* species per host species, and for coordinates of sampling sites. This resulted in data sets for *Mytilicola intestinalis* and for *Mytilicola orientalis* (available upon request) which were used for plotting occurrence maps with the R packages rworldmap^67^ and ggplot^68^. If latitude and longitude were not reported in a publication, we inferred approximate coordinates from maps so that the data could be plotted.

A possible caveat in the literature survey is that *M*. *orientalis’* invasion in Europe has been cryptic because of difficulties with reliable species identification^69^. In the literature survey, we used the identification presented by the authors, but that may be incorrect when studies are based on morphology, as the distinguishing characteristics for *M*. *intestinalis* and *M*. *orientalis* are not fully reliable^69^. For instance, *M*. *intestinalis* has been reported from *Crassostrea gigas*^70,71^, although it was shown not to be a suitable host for *M*. *intestinalis*^72,73^; these observations are more likely to be of *M*. *orientalis* instead.

### Sampling of host and parasite species

Different mussel species (*Mytilus edulis*, *Mytilus galloprovincialis*, *Mytilus trossulus* and *Brachidontes pharaonis*), and Pacific oysters (*Crassostrea gigas*) were collected in the intertidal zone during low tide at 61 locations along European coasts (Mediterranean Sea Basins, Atlantic, North Sea, and Baltic Sea), along American and Canadian Pacific coasts (Puget Sound, Vancouver Island) and along Japanese coasts (Seto Inland Sea, Sea of Japan, and Oyashio Current; Table 1). Mussel species were identified based on morphology only. There are no reports of the mussel species, *B*. *pharaonis*, being dissected for *Mytilicola* spp. Nevertheless, we included a sample from one location because if it is present in this species, this could extend the occurrence range of the parasite. Bivalves were dissected in the laboratory under a dissecting microscope (10–30×) or with the help of a magnifying glass (10×) in order to screen for the presence of *Mytilicola* individuals. Parasites were isolated from their host and stored separately by individual host in 100% ethanol.

### DNA extraction, amplification and sequencing

DNA was extracted from individual *Mytilicola* using either the QIAgen 96 DNeasy Blood & Tissue kit (Qiagen, Hilden, Germany) or the Zymo Research genomic DNA kit (the latter by Baseclear B.V., Leiden, the Netherlands). One to five *M*. *intestinalis* and one to eight *M*. *orientalis* per host were sequenced. All extractions were performed according to the protocols provided by the manufacturers. DNA concentrations were measured on a Nanodrop to confirm DNA quality and quantity. A 534 base pair (bp) fragment of the mitochondrial cytochrome-*c*-oxidase I region (COI) was amplified using the primer pair MOICOIf and MOICOIr for *M*. *intestinalis*^69^ and a newly developed primer pair for *M*. *orientalis* (MoriCOI50f: 5′-TTG ATC GGG CTT AAT TGG-3′ and MoriCOI50r: 5′-GAT CGG TTA ARA GCA TGG T-3′). The *M*. *orientalis* primers were developed in CLC Genomics Workbench 8.5.1 (CLCbio, Denmark, https://www.qiagenbioinformatics.com/) and were based on *M*. *orientalis* COI sequences from Elsner *et al*.^74^.

For *M*. *intestinalis*, polymerase chain reaction (PCR) and Sanger sequencing using both forward and reverse primers were performed according to Goedknegt *et al*.^69^ at Baseclear B.V. (Leiden, Netherlands). For *M*. *orientalis*, PCR was performed with HotStarTaq (Qiagen, Hilden, Germany) in 30 μl reaction volumes, adding 15 μl HotStarTaq Mastermix, 1.5 μl of each 10 μM primer, 11 μl H_2_O and 1 μl of sample for each reaction. Amplification started with a heat-activation of 15 min at 95 °C, followed by 38 cycles of 95 °C for 30 s, annealing at 50 °C for 30 s and elongation at 72 °C for 60 s. The final extension step was at 72 °C for 10 min. Amplification success of all *M*. *orientalis* samples was checked with a QIAxcel electrophoresis system (Qiagen, Hilden, Germany). PCR purification and Sanger sequencing of *M*. *orientalis* samples were performed on both the forward and reverse strand at the Institute of Clinical Molecular Biology (Kiel, Germany).

### Data analyses

We report parasite prevalence as the number of individual mussels or oysters infected with *M*. *intestinalis* or *M*. *orientalis* divided by the total number of mussels or oysters examined per location, and mean intensity as the mean number of *M*. *intestinalis* or *M*. *orientalis* within an infected mussel or oyster per location (*sensu* Margolis *et al*.^75^). Part of the *M*. *orientalis* sample from Tavira (Portugal) was acquired through an anti-*Mytilicola* treatment of mussels using Dichlorvos Pestanal (DDVP, 30 mg per 1 liter of seawater)^13,76^. Fifteen live *Mytilicola* specimens escaped the mussels during the recovery period shortly after the 4 h treatment. Prevalence and mean intensity (Table 1) for Tavira were calculated based on dissected mussels only.

Quality control of the sequences was performed in CLC Genomics Workbench 8.5.1 (for *M*. *orientalis*) or BioEdit 7.2.5^77^ (for *M*. *intestinalis*), after which forward and reverse reads were assembled. We screened all chromatograms for double peaks and at least one of the forward and reverse sequences had no double peaks at any base in the sequence. Some of the North American samples failed to amplify or sequence entirely and were not considered further. COI sequences were cropped to 483 base pairs (bp) for *M*. *intestinalis* and to 476 bp for *M*. *orientalis*. Unique haplotypes were determined using DnaSP 5 (*M*. *orientalis*)^78^ or using a custom Python 3.6 script (*M*. *intestinalis*) (GitHub: https://github.com/pluttik/collarl). Arlequin 3.5.1.2^79^ was used to calculate pairwise population comparisons (10,000 permutations), population differentiation based on the mitochondrial *F*-statistic Φ_ST_ and conventional *F*-statistics *F*_ST_ (10,000 permutations), Analyses of MOlecular VAriance (AMOVA), molecular diversity indices, Tajima’s D statistic^80^ and to perform Fu’s F_s_ tests^81,82^ for selective neutrality (10,000 simulated samples), mismatch distributions (10,000 permutations) and minimum spanning networks for both species. For neutral markers, Tajima’s D and Fu’s F_s_ can be used to detect changes in population size. Significantly negative D and F_s_ values can be interpreted as signatures of population expansion or, unless another independent marker has the same pattern, a past selective sweep^81,82^. We calculated both Φ_ST_ and *F*_ST_ to detect any signs of possible population differentiation, as well as for easier comparison to other studies that use either or both fixation indices. In our analyses we only included locations with more than 15 *Mytilicola* individuals, with the exception of calculations for minimum spanning networks, in which all individuals were included. In addition to overall AMOVAs, we also calculated AMOVAs within putative native and introduced regions for both species, and hierarchical AMOVAs for Mediterranean *M*. *intestinalis* with two designs to test whether the overall population differentiation could be attributed to different regions within the Mediterranean Sea Basins. The first design tested the Gulf of Lion (Cassis, Istres, Rouet and Bouzigues) vs. the Ligurian Sea (Livorno) vs. the Adriatic Sea (Malinska and Marjan). In the second design, the location Cassis was added to the Ligurian Sea group instead of the Gulf of Lion group. This was done to account for the possibility that the location Cassis, which is east of Marseille and a known location for genetic breaks^44–46^, belongs to the Italian group rather than to the group with more proximate locations within France. To test for differences in genetic diversity between species and regions, we fitted a linear model testing haplotype diversity (*h*) and nucleotide diversity (*π*) as a function of species (levels: *M*. *intestinalis* and *M*. *orientalis*), region (levels: putative native region, introduced region) and the interaction term species × region in the R statistical environment version 3.4.3^83^.

## Supplementary information

Online Resource 1

Online Resource 2

Supplementary Information

## Data Availability

Sequencing data is available on Genbank under the accession numbers MN334483-MN334508 for *M. orientalis* and MN334509-MN334526 for *M. intestinalis*.
