# Supplementary figures and images for: Global invasion genetics of two parasitic copepods infecting marine bivalves
Source: Sci Rep. 2019 Sep 4;9:12730. doi: 10.1038/s41598-019-48928-1 (PMC6726661; doi:10.1038/s41598-019-48928-1)

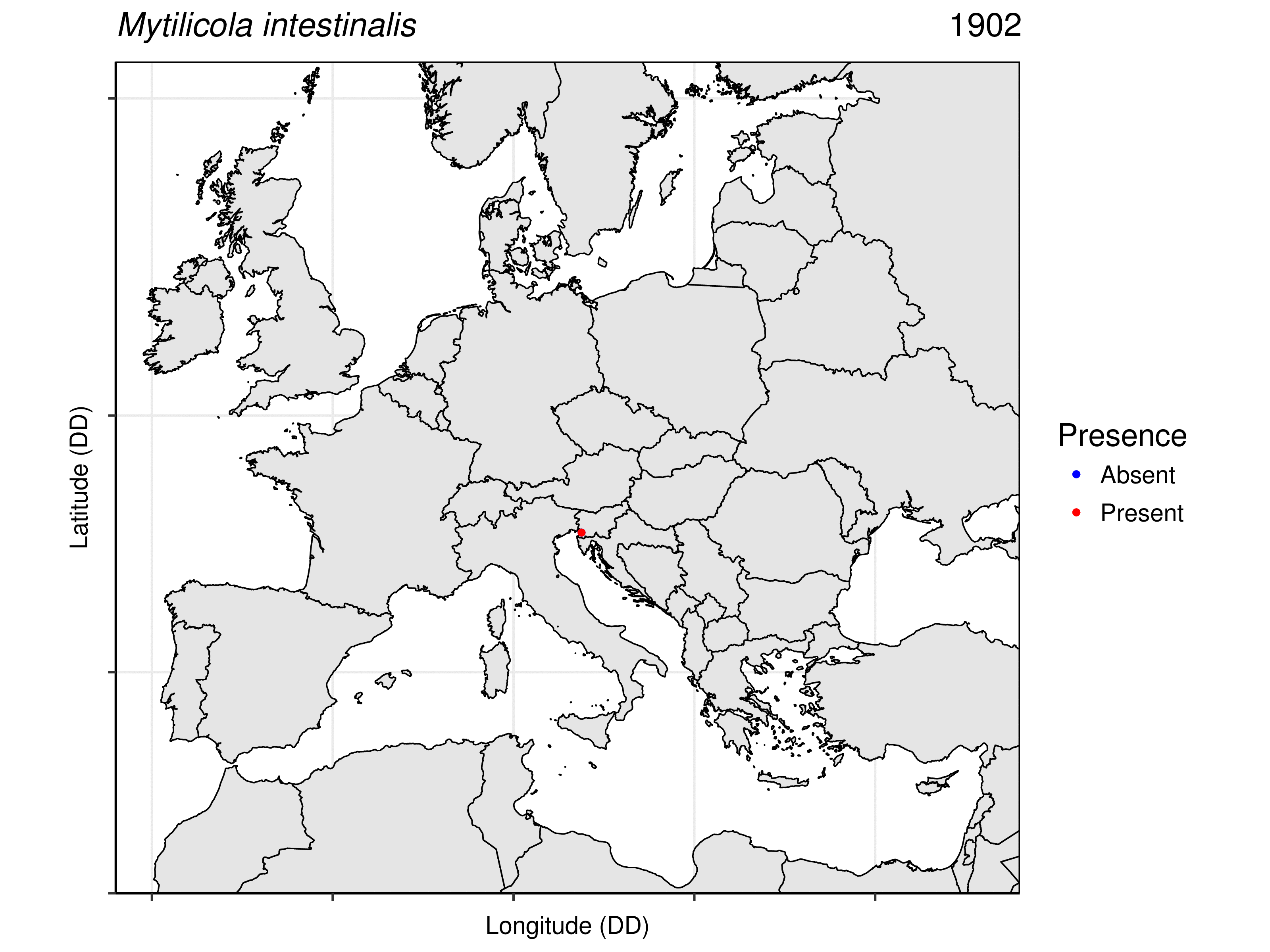

Supplement: Supplementary file 1 — Online Resource 1 [file 41598_2019_48928_MOESM1_ESM.gif]

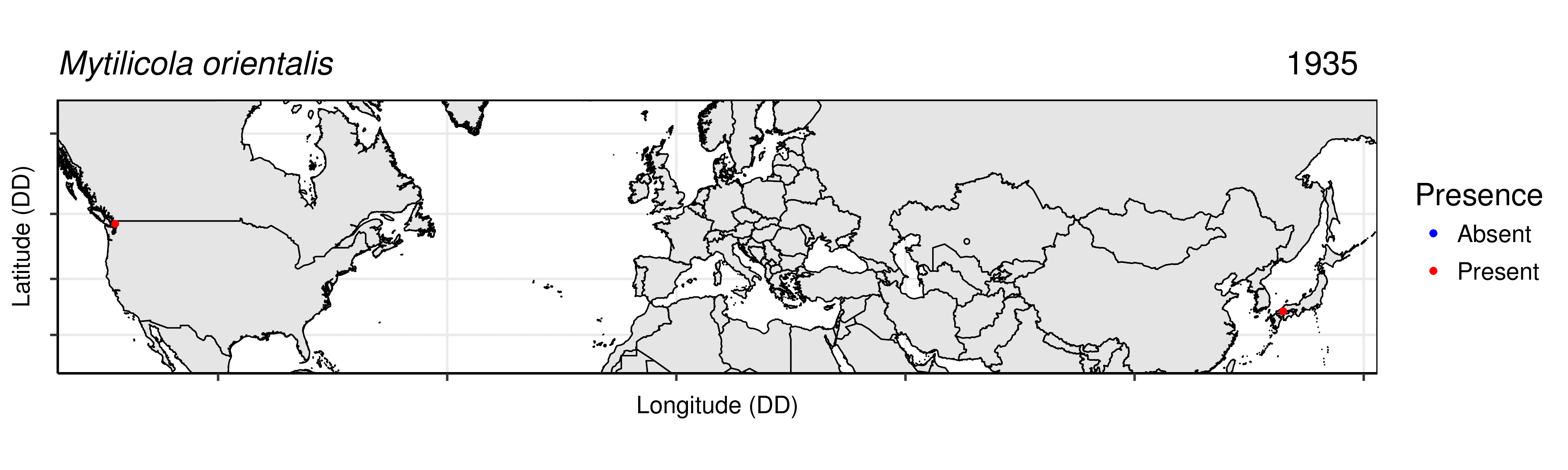

Supplement: Supplementary file 2 — Online Resource 2 [file 41598_2019_48928_MOESM2_ESM.gif]
