# Supplementary material for: Global invasion genetics of two parasitic copepods infecting marine bivalves
Source: Sci Rep. 2019 Sep 4;9:12730. doi: 10.1038/s41598-019-48928-1 (PMC6726661; doi:10.1038/s41598-019-48928-1)
Supplement: Supplementary file 3 — Supplementary Information [file 41598_2019_48928_MOESM3_ESM.pdf]

# Global invasion genetics of two parasitic copepods infecting marine bivalves

Marieke E. Feis[1,*], M. Anouk Goedknegt, Isabelle Arzul, Anne Chenuil, Onno den Boon, Leo Gottschalck, Yusuke Kondo, Susumu Ohtsuka, Lisa N. S. Shama, David W. Thieltges, K. Mathias Wegner, Pieternella C. Luttikhuizen

[1] present address: Sorbonne Université, CNRS, Station Biologique de Roscoff, Laboratoire Adaptation et Diversité en Milieu Marin, UMR 7144, CS90074, 29688 Roscoff Cedex, France

* Corresponding author: marieke.feis@sb-roscoff.fr

This Supplementary Information file contains:

Fig. S1. Cumulative *Mytilicola intestinalis* distributions per decade P. 2

Fig. S2. Cumulative *Mytilicola orientalis* distributions per decade P. 3

Fig. S3. Multidimensional scaling (MDS) plots of $\Phi_{ST}$ and $F_{ST}$ of *Mytilicola intestinalis* and *M. orientalis* P. 4

Table S1. Haplotype frequencies for 483 bp fragment of cytochrome-*c*-oxidase 1 (COI) in *Mytilicola intestinalis* P. 5

Table S2. Haplotype frequencies for 476 bp fragment of cytochrome-*c*-oxidase 1 (COI) in *Mytilicola orientalis* P. 6

Table S3. Pairwise population comparisons with the mitochondrial *F*-statistic $\Phi_{ST}$ for *Mytilicola intestinalis* P. 7

Table S4. Pairwise population comparisons with conventional *F*-statistics for *M. intestinalis* P. 8

Table S5. Pairwise population comparisons for with the mitochondrial *F*-statistic $\Phi_{ST}$ for *Mytilicola orientalis* P. 9

Table S6. Pairwise population comparisons with conventional *F*-statistics for *Mytilicola orientalis* P. 10

Reference list for *Mytilicola intestinalis* P. 11

Reference list for *Mytilicola orientalis* P. 18

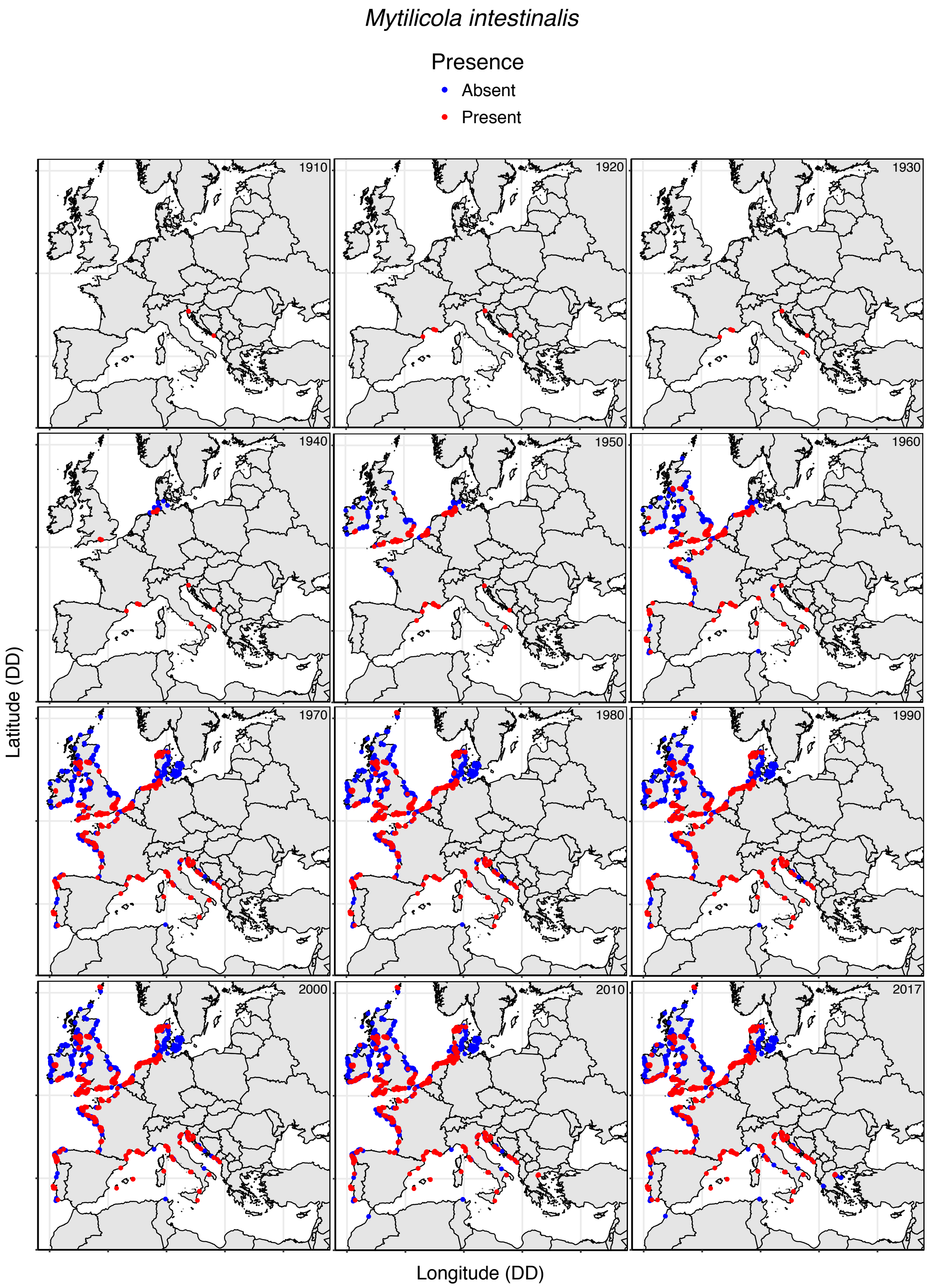


**Fig. S1.** Cumulative *Mytilicola intestinalis* distribution per decade. Cumulative year (1902 to year) is indicated in top right corner of each map and parasite presence (in red) is plotted on top of absence (in blue). Note that this figure shows both the discovery of a new occurrence (i.e., research effort) and its invasion (where blue dots appear first, then red dots). This figure is based on data from the current study and from A. Grau (pers. comm.) and on data from the literature listed in the Supplementary Information.

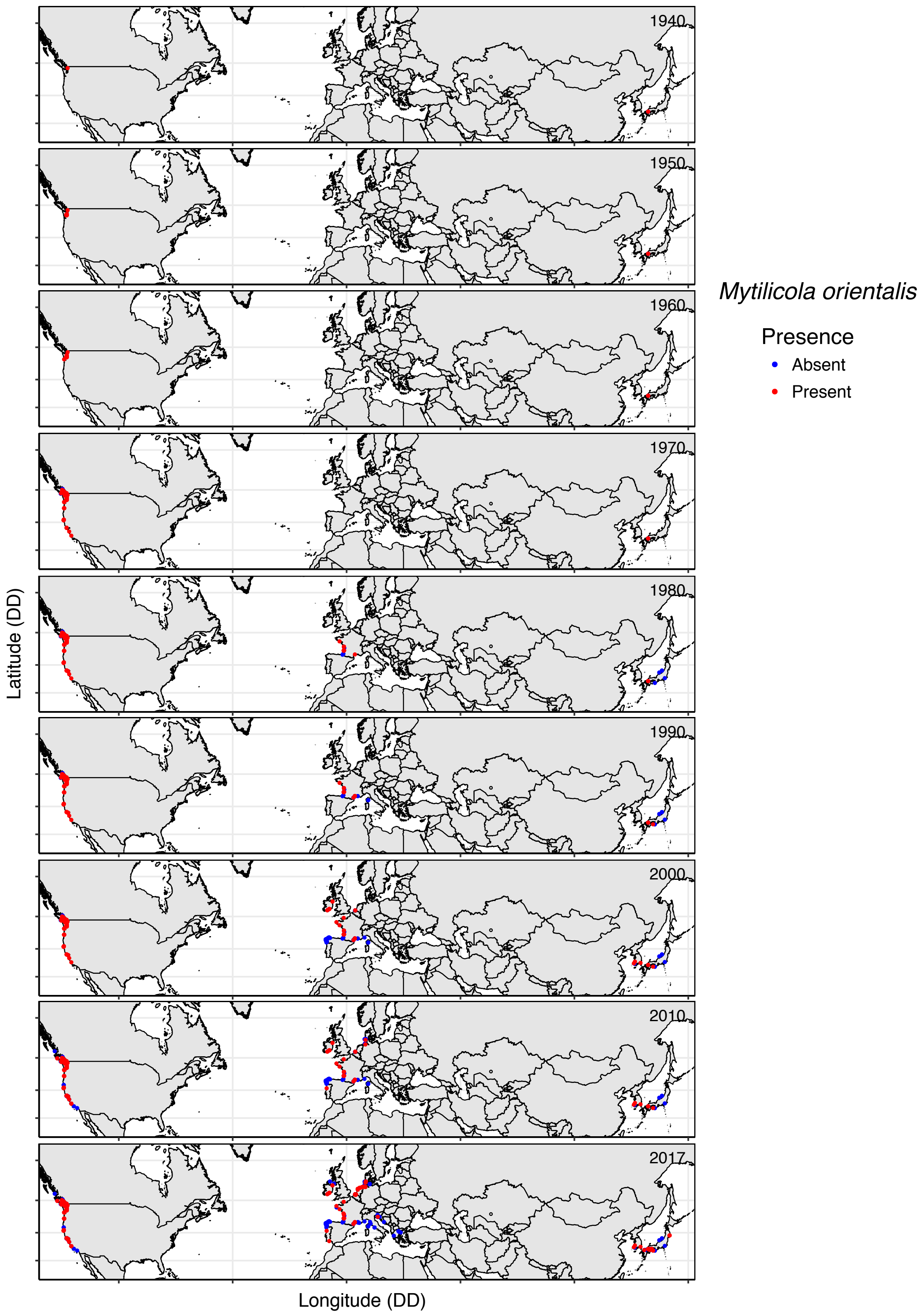


**Fig. S2.** Cumulative *Mytilicola orientalis* distribution per decade. Cumulative year (1935 to year) is indicated in top right corner of each map and parasite presence (in red) is plotted on top of absence (in blue). Note that this figure shows both the discovery of a new occurrence (i.e., research effort) and its invasion (where blue dots appear first, then red dots). This figure is based on data from the current study, from unpublished data (M. E. Feis) and data from the literature listed in the Supplementary Information.

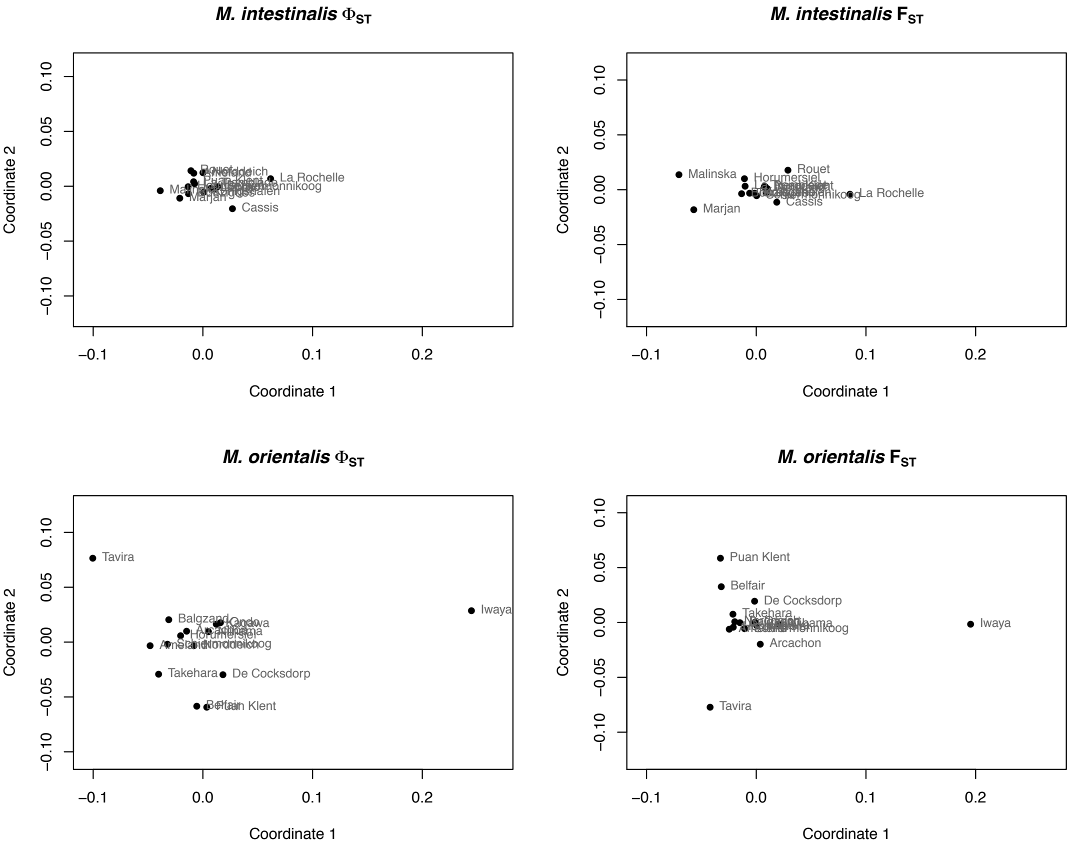


**Fig. S3.** Multidimensional scaling (MDS) plots of $\Phi_{ST}$ (mitochondrial F-statistic, left column) and $F_{ST}$ (conventional F-statistic, right column) of *Mytilicola intestinalis* (upper row) and *Mytilicola orientalis* (lower row). The overall level of population differentiation for *M. orientalis* is higher than that for *M. intestinalis*, reflected by the larger spread of locations in the lower row.

**Table S1.** Haplotype frequencies for 483 bp fragment of cytochrome-*c*-oxidase 1 (COI) in *Mytilicola intestinalis*. $\pi$: nucleotide diversity; *h*: haplotype diversity; (SD): standard deviation between brackets.

| | Mi1 | Mi2 | Mi3 | Mi4 | Mi5 | Mi6 | Mi7 | Mi8 | Mi9 | Mi10 | Mi11 | Mi12 | Mi13 | Mi14 | Mi15 | Mi16 | Mi17 | Mi18 | **Sum** | **$\pi$ (SD)** | ***h* (SD)** |
|---|---|---|---|---|---|---|---|---|---|---|---|---|---|---|---|---|---|---|---|---|---|
| Balaruc, FRA | 1 | | | | | | | | | | | | | | | | | | 1 | 0.000 (0.000) | 1.00 (0.000) |
| Marjan Peninsula, CRO | 27 | 1 | 1 | | | | | | | | | | | | | | | | 29 | 0.000286 (0.00480) | 0.1355 (0.0845) |
| Malinska, CRO | 28 | 1 | | 1 | | | | | | | | | | | | | | | 30 | 0.000276 (0.000471) | 0.131 (0.0821) |
| Livorno, ITA | 22 | 3 | 1 | | | 2 | | | | 1 | | | | | 1 | | | | 30 | 0.00134 (0.00119) | 0.460 (0.109) |
| Cassis, FRA | 22 | 1 | 4 | | | | | | | 3 | | | | | | | | | 30 | 0.00179 (0.00144) | 0.448 (0.102) |
| Istres, FRA | 25 | 1 | | | | 1 | | | 1 | 2 | | | | | | | | | 30 | 0.00121 (0.00112) | 0.308 (0.108) |
| Rouet, FRA | 19 | 3 | | 2 | | 1 | 1 | 1 | | | 1 | 1 | | | | | | | 29 | 0.00163 (0.00136) | 0.569 (0.106) |
| Bouzigues, FRA | 26 | 1 | 1 | | | | | | | | | | 1 | 1 | | | | | 30 | 0.000552 (0.000693) | 0.223 (0.104) |
| La Rochelle, FRA | 10 | | 2 | | | | | | | 1 | | | | | | 2 | 1 | | 16 | 0.00267 (0.00198) | 0.608 (0.130) |
| La Tremblade, FRA | 19 | 1 | | 1 | | | | | | | | | | | | | | 1 | 22 | 0.000753 (0.000841) | 0.260 (0.120) |
| Balgzand, NLD | 4 | | 1 | | | | | | | | | | | | | | | | 5 | 0.000828 (0.00105) | 0.4000 (0.237) |
| De Cocksdorp, NLD | 5 | | 1 | | | | | | | 1 | | | | | | | | | 7 | 0.00237 (0.00198) | 0.524 (0.209) |
| Ameland, NLD | 22 | 1 | 1 | | 1 | 3 | 1 | 1 | | | | | | | | | | | 30 | 0.00131 (0.00118) | 0.462 (0.110) |
| Schiermonnikoog, NLD | 13 | | 1 | | | 1 | | 1 | | | | | | | | | | | 16 | 0.00100 (0.00102) | 0.350 (0.148) |
| Horumersiel, DEU | 18 | 2 | | | | 1 | | | | | | | | | | | | | 21 | 0.000572 (0.000717) | 0.267 (0.120) |
| Norddeich, DEU | 33 | 1 | 2 | 2 | | 2 | | 4 | | 1 | | | | | | | | | 45 | 0.00156 (0.00130) | 0.458 (0.0898) |
| Puan Klent, DEU | 22 | 1 | 1 | 2 | | 2 | | 1 | 1 | | | | | | | | | | 30 | 0.00120 (0.00111) | 0.464 (0.111) |
| Königshafen, DEU | 19 | 2 | 2 | | | | | | | | | | | | | | | | 23 | 0.000687 (0.000795) | 0.316 (0.118) |
| Sum | **335** | **19** | **18** | **8** | **1** | **13** | **2** | **8** | **2** | **9** | **1** | **1** | **1** | **1** | **1** | **2** | **1** | **1** | **424** | | |

**Table S2.** Haplotype frequencies for 476 bp fragment of cytochrome-*c*-oxidase 1 (COI) in *Mytilicola orientalis*. $\pi$: nucleotide diversity; *h*: haplotype diversity; (SD): standard deviation between brackets.

| | Mo1 | Mo2 | Mo3 | Mo4 | Mo5 | Mo6 | Mo7 | Mo8 | Mo9 | Mo 10 | Mo 11 | Mo 12 | Mo 13 | Mo 14 | Mo 15 | Mo 16 | Mo 17 | Mo 18 | Mo 19 | Mo 20 | Mo 21 | Mo 22 | Mo 23 | Mo 24 | Mo 25 | Mo 26 | **Sum** | ***π* (SD)** | ***h* (SD)** |
|---|---|---|---|---|---|---|---|---|---|---|---|---|---|---|---|---|---|---|---|---|---|---|---|---|---|---|---|---|---|
| Iwaya, JPN | 4 | | 3 | | 3 | | | | | | | | | | 14 | | | | | | | | | | | | 24 | 0.00550 (0.00340) | 0.627 (0.092) |
| Ondo, JPN | 8 | 3 | | | 11 | 4 | | | 3 | 1 | | | | | 1 | | | | | | 1 | 1 | | | | | 33 | 0.00528 (0.00324) | 0.820 (0.0425) |
| Takehara, JPN | 6 | 3 | 2 | | 12 | 1 | | | 2 | 1 | | | | | | | | | | | | | | 1 | 1 | | 29 | 0.00422 (0.00273) | 0.788 (0.0609) |
| Niihama, JPN | 8 | 1 | 7 | | 10 | 1 | | | | 1 | | | | | 2 | | | | | | | | | | | | 30 | 0.00468 (0.00295) | 0.782 (0.0404) |
| Tarumi, JPN | 6 | 1 | 2 | | 8 | 3 | | | | | | | | | 1 | | | | | | | | | | | | 21 | 0.00436 (0.00284) | 0.776 (0.0597) |
| Ofunato, JPN | | 1 | | | | | | | | | | | | | | | | | | | | | | | | | 1 | 0.000 (0.000) | 1.00 (0.000) |
| Hiwasa, JPN | | | | | | 1 | | | | | | | | | | | | | | | | | | | | | 1 | 0.000 (0.000) | 1.00 (0.000) |
| Belfair, USA | 3 | 2 | 1 | | 10 | 2 | | | | | 1 | 1 | 1 | 1 | | | | | | | | | | | | | 22 | 0.00512 (0.00322) | 0.784 (0.0828) |
| Harstine, USA | | 1 | | | | | | | | | | | | | | | | | | | | | | | | | 1 | 0.000 (0.000) | 1.00 (0.000) |
| Cortes, CAN | 5 | | 1 | | 2 | | | | | | | | | | | | | | | | | | | | | | 8 | 0.00390 (0.00284) | 0.607 (0.164) |
| Piper’s Lagoon, CAN | 2 | | | | 3 | | | | | | | | | | | | | | | | | | | | | | 5 | 0.00378 (0.00304) | 0.600 (0.175) |
| Fanny Bay, CAN | | | | | 1 | 1 | | | | | | | | | | | | | | | | | | | | | 2 | 0.00210 (0.00297) | 1.00 (0.500) |
| Arcachon, FRA | 10 | 3 | 4 | 1 | 6 | 4 | 1 | 1 | 1 | | | | | | | | | | | | | | | | | | 31 | 0.00541 (0.00332) | 0.839 (0.0408) |
| La Rochelle, FRA | | 1 | | | | | | | | | | | | | | | | | | | | | | | | | 1 | 0.000 (0.000) | 1.00 (0.000) |
| Tavira, PRT | 16 | 2 | 2 | | 6 | 1 | | | | 3 | | | | | | | | | | | | | | | | 1 | 31 | 0.00493 (0.00307) | 0.699 (0.075) |
| Balgzand, NLD | 9 | | 3 | | 12 | 2 | | | | | | | | | 1 | 1 | 1 | | | | | | | | | | 29 | 0.00446 (0.00285) | 0.739 (0.0565) |
| De Cocksdorp, NLD | 5 | 2 | 3 | | 11 | 6 | | | 1 | | | | | | 1 | | | 1 | | | | | | | | | 30 | 0.00395 (0.00258) | 0.807 (0.0481) |
| Ameland, NLD | 10 | 2 | 3 | | 11 | 2 | | | | 2 | | | | | | | | | | | | | | | | | 30 | 0.00437 (0.00280) | 0.756 (0.0500) |
| Schiermonnikoog, NLD | 9 | 1 | 4 | | 10 | 3 | | | | 2 | | | | | | | | | | | | | 1 | | | | 30 | 0.00445 (0.00284) | 0.791 (0.0447) |
| Horumersiel, DEU | 10 | 6 | | | 11 | 3 | | | | | | | | | 1 | | | | 1 | | | | | | | | 32 | 0.00442 (0.00282) | 0.762 (0.0402) |
| Norddeich, DEU | 7 | 2 | 2 | | 10 | 5 | | | | 1 | | | | | | | | | | 1 | | | | | | | 28 | 0.00421 (0.00272) | 0.794 (0.0470) |
| Puan Klent, DEU | 3 | 2 | 2 | | 13 | 1 | | | 1 | 1 | | | | | 1 | | | | | 1 | | | | | | | 25 | 0.00366 (0.00245) | 0.723 (0.0918) |
| Sum | **121** | **33** | **39** | **1** | **150** | **40** | **1** | **1** | **8** | **12** | **1** | **1** | **1** | **1** | **22** | **1** | **1** | **1** | **1** | **2** | **1** | **1** | **1** | **1** | **1** | **1** | **444** | | |

**Table S3.** Pairwise population comparisons (below diagonal) with the mitochondrial *F*-statistic $\Phi_{ST}$ for *Mytilicola intestinalis* for all locations ($N \geq 15$), based on partial cytochrome-*c*-oxidase 1 (COI) sequences. None of the values are significantly different from 0 after Bonferroni correction (corrected *p*-level 0.05/105 = 0.000476). *P*-values are mentioned in the upper halve of the table (above diagonal).

| | Marjan | Malinska | Livorno | Cassis | Istres | Rouet | Bouzigues | La Rochelle | La Tremblade | Ameland | Schier-monnikoog | Horumersiel | Norddeich | Puan Klent | Königshafen |
|---|---|---|---|---|---|---|---|---|---|---|---|---|---|---|---|
| Marjan | - | 0.86972 | 0.58202 | 0.08336 | 0.47589 | 0.12613 | 0.99990 | 0.01030 | 0.63944 | 0.16642 | 0.20345 | 0.46184 | 0.08940 | 0.29245 | 0.53411 |
| Malinska | -0.01723 | - | 0.35224 | 0.02485 | 0.48649 | 0.12405 | 0.99990 | 0.00129 | 0.85110 | 0.10524 | 0.07732 | 0.44817 | 0.08613 | 0.41620 | 0.25542 |
| Livorno | -0.00075 | 0.00643 | - | 0.25394 | 0.77586 | 0.68221 | 0.57044 | 0.09177 | 0.50183 | 0.89021 | 0.62548 | 0.99990 | 0.35907 | 0.72834 | 0.77210 |
| Cassis | 0.04257 | 0.06074 | 0.01063 | - | 0.42085 | 0.00832 | 0.08366 | 0.66429 | 0.08930 | 0.02822 | 0.13523 | 0.09266 | 0.04208 | 0.02554 | 0.24671 |
| Istres | 0.01171 | 0.01283 | -0.01372 | -0.00116 | - | 0.10058 | 0.49282 | 0.28344 | 0.40204 | 0.27829 | 0.24423 | 0.47807 | 0.17573 | 0.41263 | 0.26483 |
| Rouet | 0.02154 | 0.01364 | -0.01100 | 0.05155 | 0.01851 | - | 0.05366 | 0.00277 | 0.43857 | 0.57291 | 0.42055 | 0.82427 | 0.23423 | 0.59954 | 0.35214 |
| Bouzigues | -0.02351 | -0.01124 | 0.00067 | 0.03969 | 0.00829 | 0.02026 | - | 0.00980 | 0.74210 | 0.17097 | 0.20998 | 0.53292 | 0.07296 | 0.28710 | 0.53599 |
| La Rochelle | 0.08404 | 0.10039 | 0.03032 | -0.02622 | 0.01087 | 0.06644 | 0.07315 | - | 0.00376 | 0.00990 | 0.20691 | 0.00535 | 0.04406 | 0.01020 | 0.07742 |
| La Tremblade | -0.00804 | -0.02057 | -0.00314 | 0.04155 | 0.00503 | -0.00245 | -0.00797 | 0.06224 | - | 0.20770 | 0.15296 | 0.59261 | 0.14909 | 0.49936 | 0.53807 |
| Ameland | 0.02229 | 0.02945 | -0.01270 | 0.04734 | 0.01557 | -0.00780 | 0.02049 | 0.06454 | 0.01580 | - | 0.96644 | 0.71023 | 0.78933 | 0.93070 | 0.28859 |
| Schiermonnikoog | 0.03356 | 0.05091 | -0.00983 | 0.02711 | 0.00964 | 0.00309 | 0.02126 | 0.03729 | 0.02218 | -0.03460 | - | 0.38105 | 0.98475 | 0.96010 | 0.23602 |
| Horumersiel | -0.00452 | -0.00286 | -0.02934 | 0.04382 | -0.00027 | -0.02097 | -0.00564 | 0.07079 | -0.01260 | -0.01487 | 0.00755 | - | 0.26730 | 0.61291 | 0.61133 |
| Norddeich | 0.02800 | 0.02928 | 0.00309 | 0.03819 | 0.01424 | 0.00824 | 0.02785 | 0.05130 | 0.01964 | -0.01255 | -0.03627 | 0.00805 | - | 0.90664 | 0.12444 |
| Puan Klent | 0.01179 | 0.00702 | -0.00627 | 0.04310 | 0.00846 | -0.00806 | 0.00851 | 0.06058 | -0.00254 | -0.01718 | -0.03253 | -0.00915 | -0.01707 | - | 0.30928 |
| Königshafen | -0.01453 | 0.01411 | -0.01532 | 0.01650 | 0.01588 | 0.00260 | -0.01150 | 0.04907 | 0.00009 | 0.00990 | 0.01711 | -0.01332 | 0.02388 | 0.00882 | - |

**Table S4.** Pairwise population comparisons with conventional *F*-statistics (below diagonal) for *Mytilicola intestinalis* for all locations (N ≥ 15), based on partial cytochrome-*c*-oxidase 1 (COI) sequences. None of the values are significantly different from 0 after Bonferroni correction (corrected *p*-level 0.05/105 = 0.000476). *P*-values are mentioned in the upper half of the table (above diagonal).

| | Marjan | Malinska | Livorno | Cassis | Istres | Rouet | Bouzigues | La Rochelle | La Tremblade | Ameland | Schier-monnikoog | Horumersiel | Norddeich | Puan Klent | Königshafen |
|---|---|---|---|---|---|---|---|---|---|---|---|---|---|---|---|
| Marjan | - | 0.99990 | 0.08336 | 0.06267 | 0.36887 | 0.01683 | 0.87130 | 0.01158 | 0.78260 | 0.07019 | 0.33017 | 0.46025 | 0.05128 | 0.08623 | 0.42273 |
| Malinska | -0.02590 | - | 0.06386 | 0.01643 | 0.36521 | 0.01733 | 0.80022 | 0.00475 | 0.71260 | 0.03930 | 0.32700 | 0.46283 | 0.04010 | 0.07821 | 0.22384 |
| Livorno | 0.04705 | 0.05387 | - | 0.55658 | 0.58608 | 0.69508 | 0.28661 | 0.30512 | 0.29957 | 0.96099 | 0.70211 | 0.58499 | 0.62608 | 0.91912 | 0.60925 |
| Cassis | 0.06190 | 0.07884 | -0.00645 | - | 0.29849 | 0.18998 | 0.14276 | 0.52341 | 0.17533 | 0.36056 | 0.50312 | 0.18681 | 0.35353 | 0.39432 | 0.48787 |
| Istres | 0.00504 | 0.00710 | -0.00734 | 0.00774 | - | 0.11949 | 0.83210 | 0.09346 | 0.83190 | 0.42402 | 0.87061 | 0.78022 | 0.30165 | 0.52124 | 0.57014 |
| Rouet | 0.08857 | 0.08707 | -0.01464 | 0.01464 | 0.02475 | - | 0.06069 | 0.27730 | 0.16979 | 0.59054 | 0.35798 | 0.23443 | 0.51074 | 0.77774 | 0.21117 |
| Bouzigues | -0.01791 | -0.01028 | 0.01022 | 0.02317 | -0.01371 | 0.04224 | - | 0.03742 | 0.99990 | 0.21265 | 0.84655 | 0.84249 | 0.15662 | 0.26334 | 0.64162 |
| La Rochelle | 0.13942 | 0.15553 | 0.00697 | -0.01446 | 0.04931 | 0.00416 | 0.07164 | - | 0.06257 | 0.29255 | 0.36878 | 0.05762 | 0.23800 | 0.30809 | 0.17196 |
| La Tremblade | -0.01370 | -0.02030 | 0.00300 | 0.02632 | -0.01914 | 0.02294 | -0.02511 | 0.06735 | - | 0.24354 | 0.67993 | 0.84992 | 0.26968 | 0.42402 | 0.69775 |
| Ameland | 0.05032 | 0.05690 | -0.02174 | 0.00327 | -0.00159 | -0.01003 | 0.01312 | 0.00868 | 0.00788 | - | 0.93753 | 0.45095 | 0.84101 | 0.98446 | 0.41135 |
| Schiermonnikoog | 0.00811 | 0.02102 | -0.02080 | -0.01163 | -0.02403 | 0.0015 | -0.02230 | 0.01075 | -0.01984 | -0.03405 | - | 0.79091 | 0.94694 | 0.91694 | 0.75438 |
| Horumersiel | -0.00796 | -0.00559 | -0.01572 | 0.02466 | -0.02357 | 0.01574 | -0.02255 | 0.06644 | -0.03049 | -0.00605 | -0.02405 | - | 0.25552 | 0.43916 | 0.73141 |
| Norddeich | 0.04708 | 0.04982 | -0.00846 | 0.00078 | 0.00383 | -0.00602 | 0.01530 | 0.00970 | 0.00667 | -0.01593 | -0.03100 | 0.00652 | - | 0.97842 | 0.37125 |
| Puan Klent | 0.04670 | 0.04651 | -0.01927 | 0.00076 | -0.00458 | -0.01688 | 0.00994 | 0.00638 | -0.00318 | -0.02432 | -0.03167 | -0.00492 | -0.02181 | - | 0.48480 |
| Königshafen | -0.00056 | 0.01598 | -0.01563 | -0.00828 | -0.01099 | 0.013 | -0.02170 | 0.02993 | -0.01914 | -0.00374 | -0.02934 | -0.02774 | -0.00002 | -0.00665 | - |

**Table S5.** Pairwise population comparisons (below diagonal) with the mitochondrial *F*-statistic $\Phi_{ST}$ for *Mytilicola orientalis* for all locations ($N \geq 15$), based on partial cytochrome-*c*-oxidase 1 (COI) sequences. Values in bold are significantly different from 0 after Bonferroni correction (corrected *p*-level 0.05/105 = 0.000476). *P*-values are mentioned in the upper half of the table (above diagonal).

| | Iwaya | Ondo | Takehara | Niihama | Tarumi | Belfair | Arcachon | Tavira | Balgzand | De Cocksdorp | Ameland | Schier-monnikoog | Horumersiel | Norddeich | Puan Klent |
|---|---|---|---|---|---|---|---|---|---|---|---|---|---|---|---|
| Iwaya | - | 0.00010 | 0.00000 | 0.00040 | 0.00129 | 0.00010 | 0.00000 | 0.00000 | 0.00010 | 0.00020 | 0.00000 | 0.00000 | 0.00000 | 0.00000 | 0.00000 |
| Ondo | **0.21472** | - | 0.54351 | 0.30720 | 0.80992 | 0.10316 | 0.64598 | 0.03871 | 0.28215 | 0.41758 | 0.49114 | 0.52500 | 0.60638 | 0.77250 | 0.29601 |
| Takehara | **0.28984** | -0.00914 | - | 0.38551 | 0.40511 | 0.24889 | 0.44827 | 0.01881 | 0.10870 | 0.39867 | 0.41630 | 0.56252 | 0.26750 | 0.51797 | 0.72300 |
| Niihama | **0.23132** | 0.00288 | -0.00138 | - | 0.79982 | 0.07920 | 0.69399 | 0.03930 | 0.40055 | 0.35016 | 0.58766 | 0.84269 | 0.23008 | 0.52470 | 0.23018 |
| Tarumi | 0.22019 | -0.02347 | -0.00603 | -0.02662 | - | 0.17266 | 0.91793 | 0.09266 | 0.67825 | 0.62687 | 0.72448 | 0.88635 | 0.72933 | 0.99515 | 0.28512 |
| Belfair | **0.26448** | 0.03027 | 0.01151 | 0.03745 | 0.02630 | - | 0.07306 | 0.00238 | 0.02455 | 0.23661 | 0.05752 | 0.09920 | 0.06752 | 0.17315 | 0.38323 |
| Arcachon | **0.25426** | -0.01257 | -0.00547 | -0.01590 | -0.02924 | 0.03954 | - | 0.16503 | 0.73250 | 0.25641 | 0.98852 | 0.98921 | 0.63231 | 0.76171 | 0.13444 |
| Tavira | **0.34408** | 0.05878 | 0.09423 | 0.06798 | 0.05197 | 0.15717 | 0.02343 | - | 0.21236 | 0.00297 | 0.21612 | 0.10435 | 0.08098 | 0.02930 | 0.00446 |
| Balgzand | **0.27312** | 0.00562 | 0.02793 | -0.00511 | -0.02307 | 0.07534 | -0.01820 | 0.00972 | - | 0.07079 | 0.77299 | 0.64330 | 0.37986 | 0.33165 | 0.05108 |
| De Cocksdorp | **0.22833** | -0.00317 | -0.00258 | 0.00182 | -0.01600 | 0.01234 | 0.00917 | 0.14485 | 0.04530 | - | 0.12524 | 0.24186 | 0.19335 | 0.78170 | 0.87298 |
| Ameland | **0.29231** | -0.00733 | -0.00364 | -0.01286 | -0.02374 | 0.05192 | -0.02780 | 0.01504 | -0.02211 | 0.02707 | - | 0.99990 | 0.49500 | 0.53747 | 0.11078 |
| Schiermonnikoog | **0.27568** | -0.00916 | -0.01038 | -0.02205 | -0.02943 | 0.03976 | -0.02715 | 0.03632 | -0.01740 | 0.00881 | -0.03053 | - | 0.41214 | 0.75725 | 0.18860 |
| Horumersiel | **0.26147** | -0.01226 | 0.00745 | 0.01074 | -0.02401 | 0.04535 | -0.01472 | 0.04482 | -0.00192 | 0.01684 | -0.01055 | -0.00480 | - | 0.55787 | 0.09039 |
| Norddeich | **0.24957** | -0.01836 | -0.00875 | -0.01023 | -0.03610 | 0.02244 | -0.01899 | 0.07688 | 0.00226 | -0.01995 | -0.01184 | -0.02033 | -0.01404 | - | 0.39442 |
| Puan Klent | **0.25493** | 0.00562 | -0.01608 | 0.01176 | 0.00891 | 0.00292 | 0.02503 | 0.16660 | 0.07160 | -0.02253 | 0.03825 | 0.01993 | 0.03638 | -0.00248 | - |

**Table S6.** Pairwise population comparisons with conventional *F*-statistics (below diagonal) for *Mytilicola orientalis* for all locations (N ≥ 15), based on partial cytochrome-*c*-oxidase 1 (COI) sequences. Values in bold are significantly different from 0 after Bonferroni correction (corrected *p*-level 0.05/105 = 0.000476). *P*-values are mentioned in the upper half of the table (above diagonal).

| | Iwaya | Ondo | Takehara | Niihama | Tarumi | Belfair | Arcachon | Tavira | Balgzand | De Cocksdorp | Ameland | Schier-monnikoog | Horumersiel | Norddeich | Puan Klent |
|---|---|---|---|---|---|---|---|---|---|---|---|---|---|---|---|
| Iwaya | - | 0.00000 | 0.00000 | 0.00000 | 0.00040 | 0.00000 | 0.00000 | 0.00000 | 0.00000 | 0.00000 | 0.00000 | 0.00000 | 0.00000 | 0.00000 | 0.00000 |
| Ondo | **0.19202** | - | 0.82170 | 0.16771 | 0.86338 | 0.54381 | 0.38293 | 0.03455 | 0.42986 | 0.72468 | 0.60766 | 0.58004 | 0.71706 | 0.87486 | 0.24720 |
| Takehara | **0.21599** | -0.01664 | - | 0.37036 | 0.77735 | 0.89219 | 0.16474 | 0.01970 | 0.65300 | 0.56796 | 0.70745 | 0.60737 | 0.49470 | 0.66439 | 0.84368 |
| Niihama | **0.16504** | 0.01624 | 0.00021 | - | 0.69468 | 0.19463 | 0.36056 | 0.04613 | 0.62786 | 0.32135 | 0.69894 | 0.85229 | 0.12425 | 0.37937 | 0.17177 |
| Tarumi | **0.19066** | -0.02274 | -0.02010 | -0.01810 | - | 0.68657 | 0.58885 | 0.09791 | 0.97832 | 0.91466 | 0.94426 | 0.96674 | 0.66766 | 0.99990 | 0.40956 |
| Belfair | **0.23027** | -0.00744 | -0.02325 | 0.01620 | -0.01781 | - | 0.07079 | 0.00495 | 0.43233 | 0.74795 | 0.35541 | 0.34967 | 0.32838 | 0.64172 | 0.97099 |
| Arcachon | **0.18733** | 0.00065 | 0.01700 | 0.00202 | -0.0114 | 0.03562 | - | 0.24017 | 0.23493 | 0.28670 | 0.49084 | 0.65726 | 0.32828 | 0.52797 | 0.01455 |
| Tavira | **0.24658** | 0.04757 | 0.06796 | 0.04657 | 0.03698 | 0.10844 | 0.01093 | - | 0.05930 | 0.00594 | 0.20533 | 0.15177 | 0.07920 | 0.04584 | 0.00168 |
| Balgzand | **0.20750** | -0.00287 | -0.01125 | -0.01293 | -0.03421 | -0.00313 | 0.01080 | 0.04260 | - | 0.41788 | 0.91229 | 0.86942 | 0.32512 | 0.67647 | 0.29472 |
| De Cocksdorp | **0.19559** | -0.01304 | -0.00853 | 0.00580 | -0.02839 | -0.01729 | 0.00749 | 0.08844 | -0.00218 | - | 0.41144 | 0.58212 | 0.28888 | 0.96268 | 0.39739 |
| Ameland | **0.21723** | -0.00957 | -0.01487 | -0.01328 | -0.03137 | 0.00188 | -0.00505 | 0.01362 | -0.02431 | 0.00078 | - | 0.99990 | 0.58559 | 0.81249 | 0.21830 |
| Schiermonnikoog | **0.20231** | -0.00812 | -0.01009 | -0.01958 | -0.03273 | 0.00275 | -0.01167 | 0.02028 | -0.02219 | -0.00837 | -0.02989 | - | 0.40115 | 0.89011 | 0.18662 |
| Horumersiel | **0.21377** | -0.01385 | -0.00600 | 0.02378 | -0.01733 | 0.00375 | 0.00256 | 0.03500 | 0.00357 | 0.00651 | -0.01088 | -0.00049 | - | 0.59727 | 0.12345 |
| Norddeich | **0.21300** | -0.01996 | -0.01297 | 0.00061 | -0.03724 | -0.01420 | -0.00640 | 0.04774 | -0.01501 | -0.02632 | -0.02032 | -0.02380 | -0.01150 | - | 0.31225 |
| Puan Klent | **0.23398** | 0.00918 | -0.02052 | 0.01990 | -0.00132 | -0.02811 | 0.06490 | 0.13514 | 0.00544 | -0.00009 | 0.01426 | 0.01728 | 0.02723 | 0.00551 | - |

## Reference list for *Mytilicola intestinalis*

Data from papers cited in this list underlie plotting of the *Mytilicola intestinalis* distributions in Fig. 1, Supplementary Fig. S1, and Online Resource 1.


1. Aguirre-Macedo, M. L. & Kennedy, C. R. Diversity of metazoan parasites of the introduced oyster species *Crassostrea gigas* in the Exe Estuary. *J. Mar. Biol. Ass. U.K.* **79,** 57–63 (1999).
2. Aguirre-Macedo, M. I. & Kennedy, C. R. Patterns in metazoan parasite communities of some oyster species. *J. Helminthol.* **73,** 283–288 (1999).
3. Ahrens, W. Welche Tatsachen können zur Beurteilung der Meiosis als gesichtert vorausgesetzt werden? (Eine Erwidering an G. Heberer und zugleich eine vorläufige Mitteilung über die Grundzüge der Meiosis von *Mytilicola*). *Zool. Anz.* **120,** 241–267 (1937).
4. Ahrens, W. Die Entwicklung des primären Spaltes der Copepoden-Tetraden nach Untersuchungen über die Meiose (Oogenese) von *Mytilicola intestinalis*. *Zeitschr. Mikrosk.-anat. Forsch.* **46,** 68–120 (1939).
5. Andreu, B. Dispersión de *Mytilicola intestinalis* Steuer en el mejillón cultivo a flote de la rías de Arosa y Vigo (NW de España). *Reun. Prod. Pesq.* **4,** 115–118 (1960).
6. Andreu, B. Un parásito del mejillón. Propagación del copépodo parásito *Mytilicola intestinalis* en el mejillón de las rías bajas. *Rev. Econ. Galicia* **17–18,** 12–18 (1961).
7. Andreu, B. Propagación del copépodo parásito *Mytilicola intestinalis* en el mejillón cultivado de las rías gallegas (NW de España). *Inv. Pesq.* **24,** 3–20 (1963).
8. Andreu, B. Biología y parasitologia del mejillón gallego. *Las Ciencas* **30,** 107–118 (1965).
9. Andreu, B. & Ezama, A. *Consideraciones sobre la presencia de* Mytilicola intestinalis *en el mejillón de las rias gallegas*. (1953).
10. Anonymous. Rapport sur l'activite de l'Institut Scientifique et Technique des Pêches Maritimes. (1962).
11. Auffret, M. & Poder, M. Pathology of the main bivalve mollusc species from oyster rearing areas in Brittany (France). *Aquaculture* **67,** 255–257 (1987).
12. Bacci, G., Balatu, M. & Romani, M. L. Rapporti numerici dei sessi in tre popolazioni di *Mytilicola intestinalis* Steuer. *Rend. Accad. Naz. Lincei* **25,** 557–563 (1958).
13. Baird, R. H., Bolster, G. C. & Cole, H. A. *Mytilicola intestinalis*, Steuer, in the European flat oyster (*Ostrea edulis*). *Nature* **168,** 560 (1951).
14. Bassedas, M. Sobre la presencia de *Mytilicola intestinalis* Steuer en Barcelona. *P. Inst. Biol. Appl.* **7,** 153–154 (1950).
15. Bignell, J. P. *et al.* Mussel histopathology: effects of season, disease and species. *Aquat. Biol.* **2,** 1–15 (2008).
16. Bignell, J. P., Stentiford, G. D., Taylor, N. G. & Lyons, B. P. Histopathology of mussels (*Mytilus* sp.) from the Tamar estuary, UK. *Mar. Environ. Res.* **72,** 25–32 (2011).
17. Blateau, D. Expériences de traitement des moules (*M. edulis*) de bouchots de la baie du mont sain-tmichel parasitées par *Mytilicola intestinalis*. *IFREMER Rep. n° 80/ 210779/NR* (1989).
18. Blateau, D., Le Coguic, Y., Mailhe, E. & Grizel, H. Mussel (*Mytilus edulis*) treatment against the red copepod *Mytilicola intestinalis*. *Aquaculture* **107,** 165–169 (1992).
19. Bocquet, C. & Stock, J. H. Copépodes parasites d'invertébrés des côtes de France. II & III Notes taxonomiques et écologiques sur la famille des Mytilicolidae. Travaux de la Station Biologique de Roscoff, 8. *Proc. Acad. Sci. Amst., Séries C* **60,** 223–239 (1957).
20. Bolster, G. C. The biology and dispersal of *Mytilicola intestinalis* Steuer: a copepod parasite of mussels. *Fish. Investig. London* (1954).
21. Boyden, C. R., Crothers, J. H., Little, C. & Mettam, C. The intertidal invertebrate fauna of the Severn Estuary. *Fld Stud.* **4,** 477–554 (1977).
22. Brégeon, L. Richesses et productions marines de la Baie du Mont-Saint-Michel: La Mytiliiculture. *Sci. Pêche* **267,** 2–29 (1977).
23. Brenner, M. Assessing the health of blue mussels (*Mytilus edulis*) for site-selection of cultivation areas: potentials and constraints of applied parameters. *ICES C.* **F:13,** (2010).
24. Brienne, H. *Mytilicola intestinalis* (Steuer) dans les moules de la baie de l'Aiguillon. *Sci. Pêche* **87,** 1–6 (1960).
25. Brienne, H. Evolution de l'infestation des moules de la baie de l'Aiguillon par *Mytilicola intestinalis* (Steuer) au cours de l'année 1962. *Sci. Pêche* **106,** 1–5 (1962).
26. Brienne, H. Observations sur l'infestation des moules du Pertuis Breton par *Mytilicola intestinalis* (Steuer). *Rev. Trav. Off. Sci. Tech. Pêches Marit.* **28,** 205–230 (1964).

27. Buck, B. H., Thieltges, D. W., Walter, U., Nehls, G. & Rosenthal, H. Inshore-offshore comparison of parasite infestation in *Mytilus edulis*: implications for open ocean aquaculture. *J. Appl. Ichthyol.* **21,** 107–113 (2005).
28. Cabral, H. & Wouters, N. *Relação entre a comunidade de macroparasitas e indicadores parasitológicos, e sua influência no sistema lagunar*. (2013).
29. Campbell, S. A. Seasonal cycles in the carotenoid content in *Mytilus edulis*. *Mar. Biol.* **4,** 227–232 (1969).
30. Campbell, S. A. The occurrence and effects of *Mytilicola intestinalis* in *Mytilus edulis*. *Mar. Biol.* **5,** 89–95 (1970).
31. Canestri Trotti, G., Baccarani, E. M., Giannetto, S., Giuffrida, A. & Paesanti, F. Prevalence of *Mytilicola intestinalis* (Copepoda: Mytilicolidae) and *Urastoma cyprinae* (Turbellaria: Hypotrichinidae) in marketable mussels *Mytilus galloprovincialis* in Italy. *Dis. Aquat. Organ.* **32,** 145–149 (1998).
32. Carballal, M. J., Iglesias, D., Santamarina, J., Ferro-Soto, B. & Villalba, A. Parasites and pathologic conditions of the cockle *Cerastoderma edule* populations of the coast of Galicia (NW Spain). *J. Invert. Path.* **78,** 87–97 (2001).
33. Carballal, M. J., Villalba, A. & López, C. Seasonal variation and effects of age, food availability, size, gonadal development, and parasitism on the hemogram of *Mytilus galloprovincialis*. *J. Invert. Path.* **72,** 304–312 (1998).
34. Caspers, H. Über Vorkommen und Metamorphose von *Mytilicola intestinalis* Steuer (Copepoda paras.) in der südlichen Nordsee. *Zool. Anz.* **126,** 161–171 (1939).
35. Cerruti, A. La pesea nei mari e nelle aeque interne d'Italia. *Molluschicoltura* **2,** 365–391 (1932).
36. Ceschia, G., Mion, A., Orel, G. & Giorgetti, G. Indagine parassitologicadelle mitilicolture del Friuli-Venezia Giulia (Nord-Est Italia). *Boll. Soc. Ital. Patol. Ittica* **9,** 24–36 (1992).
37. Cole, H. A. Le *Mytilicola* en Angleterre. *Rev. Trav. Off. Sci. Tech. Pêches Marit.* **17,** 59–61 (1951).
38. Cole, H. A. & Savage, R. E. The effect of the parasitic copepod, *Mytilicola intestinalis* (Steuer) upon the condition of mussels. *Parasitology* **41,** 156–161 (1951).
39. Comps, M. & Pichot, Y. *Situation zoosanitaire des coquillages en Mediterranée (1985-1987). Rapports internes de la Direction des Ressources Vivantes de l'IFREMER* (1989).
40. Cormaci, M. The presence of *Mytilicola intestinalis* (Crustacea Copepoda) in cultures of *Mytilus galloprovincialis* near Siracusa, Italy. *Atti Accad. Gioenia Sci. Nat.* **7,** 51–62 (1973).
41. Costanzo, G. Sullo sviluppo di *Mytilicola intestinalis* Steuer (Crost. Cop.). *Arch. Zool. Ital.* **44,** 151–163 (1959).
42. Coste, F., Manier, J.-F. & Raibout, A. Un type structural de spermatozoide chez les copépodes. *Crustaceana* **43,** 249–260 (1982).
43. Couteaux-Bargeton, M. Contribution à l'etude de *Mytilus edulis* L. parasité par *Mytilicola intestinalis* Steuer. *J. Cons. Int. Pour l'Exploitation la Mer* **19,** 80–84 (1953).
44. Crowley, M. The parasitology of Irish mussels (*Mytilus edulis* L.). *Fish. Leafl.* **35,** (1972).
45. Crowley, M. The parasitology of Irish mussels (*Mytilus edulis* L.). *Fish. Leafl.* **75,** 11 pages (1975).
46. Dardignac-Corbeil, M. J. *La mytiliculture dans le" Pertuis Breton": synthèse des travaux réalises de 1980 a 1992. Annales de la Société des sciences naturelles de la Charente-Maritime* (Société des sciences naturelles de la Charente-Maritime, 2004).
47. Dardignac-Corbeil, M. J. & Feuillet, M. Croissance des moules de bouchots dans la baie de l'Aiguillon (premières observations). *Cons. int. Explor. mer, Com. Crust., Coquill. Benth., C.* **K:34,** (1974).
48. Dare, P. J. A survey of the distribution limits of *Mytilicola intestinalis* Steuer in England and Wales, 1972-1974. *ICES Counc. Meet. Shellfish Comm.* **K:12,** (1974).
49. Davey, J. T. *Mytilicola intestinalis* (Copepoda: Cyclopoida): a ten year survey of infested mussels in a Cornish estuary, 1978-1988. *J. Mar. Biol. Ass. U.K.* **69,** 823–836 (1989).
50. Davey, J. T. & Gee, J. M. The occurrence of *Mytilicola intestinalis* Steuer, an intestinal copepod parasite of *Mytilus*, in the South-West of England. *J. Mar. Biol. Ass. U.K.* **56,** 85–94 (1976).
51. de Montaudouin, X., Kisielewski, I., Bachelet, G. & Desclaux, C. A census of macroparasites in an intertidal bivalve community, Arcachon Bay, France. *Oceanol. Acta* **23,** 453–468 (2000).
52. Dethlefsen, V. On the parasitology of *Mytilus edulis* L. *ICES Counc. Meet. Shellfish Comm.* **K:16,** 11 pp (1970).
53. Dethlefsen, V. Zur Parasitologie der Miesmuschel (*Mytilus edulis* L., 1758). *Ber. dt. wiss.*

*Kommn. Meeresforsch.* **22,** 344–371 (1972).
54. Dethlefsen, V. Seasonal fluctuations in two parasitic copepods *Mytilicola intestinalis* Steuer and *Modiolicola insignis* Aurivillius. *Ber. dt. wiss. Kommn. Meeresforsch.* **23,** 376–392 (1974).
55. Dethlefsen, V. The influence of *Mytilicola intestinalis* Steuer on the meat content of the mussel *Mytilus edulis* L. *Aquaculture* **6,** 83–97 (1975).
56. Dollfus, R. P. *Trochicola enterica*, nov. gen., nov. sp., eucopépode parasite de l'intestin des troques. *C. R. Hebd. Seances Acad. Sci.* **CLVIII,** 1528–1531 (1914).
57. Dollfus, R. P. Notules sur des copépodes de la faune française I-III. *Bull. la Société Zool. Fr.* **2,** 119–121 (1927).
58. Drinkwaard, A. C. *Het gevaar van de mosselparasiet voor de mosselkweek.* (Unpublished Report, commissioned by Productschap voor Vis en Visproducten, the Netherlands, 1993).
59. Drinkwater, J. Further observations on the distribution of *Mytilicola intestinalis* around Scotland. *ICES Counc. Meet. Shellfish Comm.* **31,** (1971).
60. Drinkwater, J. & Howell, T. R. W. A method of detecting *Mytilicola intestinalis* Steuer in mussels, *Mytilus edulis* L., using pepsin digestion, and its use in a survey in Scottish waters. *ICES Counc. Meet. Shellfish Comm.* **K:28,** 3 pp (1977).
61. Durfort i Coll, M. Òrgans diana de les principals parasitosis de mol.luscs bivalves d'interès gastronòmic. *Rev. la R. Acadèmia Med. Catalunya* **17,** 97–103 (2002).
62. Durfort, M. Consideraciones sobre la estructura y ultraestructura del epitelio intestinal de *Mytilicola intestinalis*, Steuer. *I Centen. Real Soc. Esp. Hist. Nat.* **II,** 109–120 (1975).
63. Durfort, M., Bargallo, B., Bozzo, M. G., Fontarnau, R. & López-Camps, J. Alterations des ovocytes de *Mytilus edulis*, L. (Mollusca, Bivalvia) dues a l'infestation de la moule par *Mytilicola intestinalis*, Steuer (Crustacea, Copepoda). *Malacologia* **22,** 55–59 (1982).
64. Durfort, M., Bargallo, R., Bozzo, M. G. & Fontarnau, R. Présence de ponts intercellulaires entre les cellules germinales de *Mytilicola intestinalis* Steuer (Crustacea, Copepoda). *Cellule* **73,** 205–213 (1980).
65. Ellenby, C. A copepod parasite of the mussel new to the British fauna. *Nature* **159,** 645 (1947).
66. Elsner, N. O., Jacobsen, S., Thieltges, D. W. & Reise, K. Alien parasitic copepods in mussels and oysters of the Wadden Sea. *Helgol. Mar. Res.* **65,** 299–307 (2010).
67. Fermer, J., Culloty, S. C., Kelly, T. C. & O'Riordan, R. M. Parasitological survey of the edible cockle *Cerastoderma edule* (Bivalvia) on the south coast of Ireland. *J. Mar. Biol. Assoc. United Kingdom* **91,** 923–928 (2011).
68. Figueras, A. & Figueras, A. J. *Mytilicola intestinalis* Steuer, in cultivated mussels in the Ria of Vigo. *Invest. Pesq. Barc.* **45,** 263–278 (1981).
69. Figueras, A. J., Jardon, C. F. & Cladras, J. R. Diseases and parasites of rafted mussels (*Mytilus galloprovincialis* Lmk): preliminary results. *Aquaculture* **99,** 17–33 (1991).
70. Francisco, C. J., Hermida, M. A. & Santos, M. J. Parasites and symbionts from *Mytilus galloprovincialis* (Lamark, 1819) (Bivalves: Mytilidae) of the Aveiro Estuary Portugal. *J. Parasitol.* **96,** 200–205 (2010).
71. Fuentes, J., Villalba, A., Zapata, C. & Alvarez, G. Effects of stock and culture environment on infections by *Marteilia refringens* and *Mytilicola intestinalis* in the mussel *Mytilus galloprovincialis* cultured in Galicia (NW Spain). *Dis. Aquat. Organ.* **21,** 221–226 (1995).
72. Gam, M. Dynamique des systemes parasites - hote, entre trematodes digenes et coque *Cerastoderma edule*: comparaison de la lagune de Merja Zerga avec le Bassin d'Arcachon. **PhD,** (2008).
73. Garmendia, L., Soto, M., Cajaraville, M. P. & Marigómez, I. Seasonality in cell and tissue-level biomarkers in *Mytilus galloprovincialis*: relevance for long-term pollution monitoring. *Aquat. Biol.* **9,** 203–219 (2010).
74. Gee, J. M., Maddock, L. & Davey, J. T. The relationship between infestation by *Mytilicola intestinalis*, Steuer (Copepoda, Cyclopoidea) and the condition index of *Mytilus edulis* in southwest England. *J. Cons. Int. Pour l'Exploitation la Mer* **37,** 300–308 (1977).
75. Gibson, F. A. A note upon the occurrence of *Mytilicola intestinalis* (Steuer) around the Irish coast. *ICES Counc. Meet. Shellfish Comm.* **56,** (1961).
76. Giusti, F. L'azione della *Mytilicola intestinalis* Steuer sul *Mytilus galloprovincialis* Lam. del litorale Toscano. *Riv. Parassitol.* **28,** 17–26 (1967).
77. Goedknegt, M. A. Pacific oysters and parasites: Species invasions and their impact on parasite-host interactions. **PhD,** (VU University Amsterdam, 2017).
78. Goedknegt, M. A. *et al.* Spillover but no spillback of two invasive parasitic copepods from invasive Pacific oysters (*Crassostrea gigas*) to native bivalve hosts. *Biol. Invasions* **19,** 365–379 (2017).

79. Grainger, J. N. Notes on the biology of the copepod *Mytilicola intestinalis* Steuer. *Parasitology* **41,** 135–142 (1951).
80. Green, M. & Alderman, D. J. Neoplasia in *Mytilus edulis* L. from United Kingdom waters. *Aquaculture* **30,** 1–10 (1983).
81. Gresty, K. A. Ultrastructure of the midgut of the copepod *Mytilicola intestinalis* Steuer, an endoparasite of the mussel *Mytilus edulis* L. *J. Crustac. Biol.* **12,** 169–177 (1992).
82. Gresty, K. A. & Quarmby, C. The trophic level of *Mytilicola intestinalis* Steuer (Copepoda: Poecilostomatoida) in *Mytilus edulis* L., as determined from stable isotope analysis. *Bull. Plankt. Soc. Japan* **Special Vo,** 363–371 (1991).
83. Hancock, D. A. Adductor muscle size in Danish and British mussels and its relation to starfish predation. *Ophelia* **2,** 253–267 (1965).
84. Heldt, J. H. Observations sur *Mytilicola intestinalis* Steuer parasite des moules. *Rev. Trav. Off. Pech. Marit.* **17,** 33–39 (1951).
85. Hepper, B. T. Environmental factors governing the infection of mussels, *Mytilus edulis*, by *Mytilicola intestinalis*. *Min. Agric. Fish. Food, Fish. Invest., London, Ser. 2* **20,** 1–21 (1955).
86. Hepper, B. T. The European flat oyster, *Ostrea edulis* L., as a host for *Mytilicola intestinalis* Steuer. *Jounal Anim. Ecol.* **25,** 144–147 (1956).
87. Hepper, B. T. Notes on *Mytilus galloprovincialis* Lamarck in Great Britain. *J. Mar. Biol. Ass. U.K.* **36,** 33–40 (1957).
88. His, E. Mytilicolides et Myicolides parasites des lamellibranches d'intérêt commercial du Bassin d'Arcachon. *Haliotis* **8,** 99–102 (1979).
89. Hockley, A. R. On the biology of *Mytilicola intestinalis* (Steuer). *J. Mar. Biol. Ass. U.K.* **30,** 223–232 (1951).
90. Hrs-Brenko, M. *Mytilicola intestinalis* Steuer as a parasite of mussels in natural beds and artificial rearing places of the Eastern Adriatic. *Acta Adriat.* **11,** 161–165 (1964).
91. Hrs-Brenko, M. & Bozić, E. *Influence of the parasite* Mytilicola intestinalis *Steuer on mussels. Proc. and techn. Papers* **8,** (General Fisheries Council for the Mediterranean, FAO, 1965).
92. Izagirre, U., Garmendia, L., Soto, M., Etxebarria, N. & Marigómez, I. Health status assessment through an integrative biomarker approach in mussels of different ages with a different history of exposure to the Prestige oil spill. *Sci. Total Environ.* **493,** 65–78 (2014).
93. Jaafar Kefi, F., Gargouri Ben Abdallah, L., Trigui El-Menif, N., Mraouna, R. & El Bour, M. Health status of the date mussel *Lithophaga lithophaga* (Linné, 1758) from the North of Tunisia. *Cah. Biol. Mar.* **53,** 177–184 (2012).
94. Jensen, K. R. NOBANIS - Invasive Alien Species Fact Sheet - *Mytilicola intestinalis*. (2010).
95. Jungblut, S. BSc thesis. Macroparasites in Pacific oysters (*Crassostrea gigas*) of the East Frisian Waddn Sea, Germany, with focus on copepods. (2011).
96. Karagiannis, D., Vatsos, I. N., Theodoris, A. & Angelidis, P. Effect of culture system on the prevalence of parasites of the Mediterranean mussel *Mytilus galloprovincialis* (Lamark, 1819). *J. Hell. Vet. Med. Soc.* **64,** 113–122 (2013).
97. Korringa, P. De aanval van de parasiet *Mytilicola intestinalis* op de Zeeuwse mosselcultuur. *Viss. Nieuws* **7,** 1–7 (1950).
98. Korringa, P. Le *Mytilicola intestinalis* Steuer (Copepoda Parasitica) menace l'industrie moulière en Zélande. *Rev. Trav. Off. Pech. Marit.* **17,** 9–13 (1951).
99. Korringa, P. Over *Mytilicola intestinalis* (Copepoda parasitica) en enkele andere ongewenste vreemdelingen in onze wateren. *Vakbl. Biol.* **31,** 63–74 (1951).
100. Korringa, P. Epidemiological observations on the mussel parasite *Mytilicola intestinalis* Steuer, carried out in the Netherlands, 1951. *Ann. Biol. Copen.* **8,** 182–185 (1952).
101. Korringa, P. Epidemiological observations on the mussel parasite *Mytilicola intestinalis* Steuer, carried out in the Netherlands, 1952. *Ann. Biol. Copen.* **9,** 219–224 (1953).
102. Korringa, P. Epidemiological observations on the mussel parasite *Mytilicola intestinalis* Steuer, carried out in The Netherlands 1953. *Ann. Biol. Copen.* **10,** 197–200 (1954).
103. Korringa, P. Epidemiological observations on the mussel parasite *Mytilicola intestinalis* Steuer, carried out in the Netherlands 1954. *Ann. Biol. Copen.* **11,** 184–186 (1956).
104. Korringa, P. Epidemiological observations on the mussel parasite *Mytilicola intestinalis* Steuer, undertaken in the Netherlands in 1955. *Ann. Biol. Copen.* **12,** 230–231 (1957).
105. Korringa, P. Checking *Mytilicola*'s advance in the Dutch Waddensea. *Cons. Perma. Int. Explor. Mer, 47th Meet. 1959, Shellfish Comm.* **87,** 3 (1959).
106. Korringa, P. & Lambert, L. Quelques observations sur la fréquence de *Mytilicola intestinalis* Steuer (Copepoda parasitica) dans les moules du littoral méditerranéen français. *Rev. Trav. Off. Sci. Tech. Pêches Marit.* **17,** 15–29 (1951).

107. Krakau, M. Eingeschleppte und heimische Mollusken im Wattenmeer: Unterschiede in Bewuchs und Parasitierung? *Biologie/Chemie* **Diplomarbeit,** (Universität Bremen, 2004).
108. Krakau, M., Thieltges, D. W. & Reise, K. Native parasites adopt introduced bivalves of the North Sea. *Biol. Invasions* **8,** 919–925 (2006).
109. Lambert, L. Le Cop rouge (*Mytilicola intestinalis*) (Steuer) sur les côtes de France. *Rev. Trav. Off. Sci. Tech. Pêches Marit.* **17,** 51–56 (1951).
110. Le Dantec, J. & Borel, M. Les moules du littoral atlantique au sud de la Gironde et leurs crutacés parasites (observations d'octobre 1963 à avril 1964) sur la rive gauche de la Gironde et dans le Bassin d'Arcachon. *Cons. int. Explor. mer, Com. Moll. Crust.* **34,** (1964).
111. Leloup, E. Sur la présence de *Mytilicola intestinalis* Steuer le long de la côte Belge. *Rev. Trav. Off. Sci. Tech. Pêches Marit.* **17,** 57–58 (1951).
112. Leloup, E. *Mytilicola* le long de la Côte Belge. *ICES Counc. Meet. Shellfish Comm.* **37,** (1959).
113. Leloup, E. Recherches sur la repartition de *Mytilicola intestinalis* Steuer, 1905, le long de la cote Belge (1950-1958). *Bull. Inst. Roy. Sci. Natur. Belg.* **36,** 1–12 (1960).
114. López-Camps, J., Bargallo, R. & Fontarnau, R. The spermatogenesis of crustaceans. VI. The external morphology of the mature spermatozoa of *Mytilicola intestinalis*, Steuer (Copepoda). *Gamete Res.* **2,** 235–245 (1979).
115. Lynch, S. A. *et al.* The health status of mussels, *Mytilus* spp., in Ireland and Wales with the molecular identification of a previously undescribed haplosporidian. *J. Invertebr. Pathol.* **118,** 59–65 (2014).
116. Mackenzie, C. L., Lynch, S. A., Culloty, S. C. & Malham, S. K. Future oceanic warming and acidification alter immune responses and disease status in a commercial shellfish species, *Mytilus edulis* L. *PLoS One* **9,** e99712 (2014).
117. Marteil, L. Répartition actuelle de *Mytilicola intestinalis* Steuer sur la côte sud de Bretagne. *ICES Counc. Meet. Shellfish Comm.* **58,** (1955).
118. Marteil, L. *Mytilicola intestinalis* Steuer sur la côte Sud de Bretagne. *ICES Counc. Meet. Shellfish Comm.* **30,** 3 pp (1960).
119. Mason, J. The distribution of *Mytilicola intestinalis* (Steuer) in Scottish waters in 1961. *ICES, C. M.* **75,** 1–9 (1961).
120. Mason, J. & Fraser, D. I. Shellfish fisheries in the Clyde Sea Area. *Proc. R. Soc. Edinburgh* **90,** 439–450 (1986).
121. Meyer, P. F. & Mann, H. Beiträge zur Epidemiologie und Physiologie des parasitischen Copepoden *Mytilicola intestinalis*. *Arch Fischereiwiss* **2,** 120–134 (1950).
122. Meyer, P. F. & Mann, H. Recherches allemandese relatives en *Mytilicola* copepode parasite de la moule existant dans les watten allemandes, 1950-1951. *Rev. Trav. Off. Pech. Marit.* **17,** 63–71 (1951).
123. Meyer-Waarden, P. F. *Mytilicola intestinalis* in the German Waddensea. 1954. *Ann. Biol. Copen.* **11,** 184 (1956).
124. Meyer-Waarden, P. F. & Mann, H. Untersuchungen über die Bestände von *Mytilus galloprovincialis* an der italienischen Küste auf ihren Befall mit *Mytilicola intestinalis* (Copepoda parasitica). *Boll. Pesca Piscic. Idrobiol., Roma* **8,** 201–220 (1953).
125. Meyer-Waarden, P. F. & Mann, H. Der Befall von *Mytilus edulis* durch *Mytilicola intestinalis* in den deutschen Wattengebieten 1950-1953. *Ber. dt. wiss. Kommn. Meeresforsch.* **13,** 347–362 (1954).
126. Meyer-Waarden, P. F. & Mann, H. Ein weiterer Beitrag zur Epidemiologie von *Mytilicola intestinalis* (Infektionsversuche). *Arch. Fischereiwiss.* **5,** 26–31 (1954).
127. Mladineo, I. Risk assessment of parasitic/symbiotic organisms of the commercially important mytilid *Modiolus barbatus* (Linnaeus, 1758). *Aquac. Res.* **39,** 1705–1719 (2008).
128. Monteiro, M. C. & De Figeuiredo, M. J. Présence du Cop Rouge, *Mytilicola intestinalis* Steuer, dans les moules de l'estuaire du Tage. 1959. *ICES Counc. Meet. Shellfish Comm.* **113,** 7 pp (1959).
129. Monteiro, M. C. & Figeuiredo, M. J. Note sur la répartition de *Mytilicola intestinalis* Steuer dans l'estuaire du Tage. *Notas Est. Inst. Biol. Marit., Lisboa* **22,** 1–13 (1961).
130. Moreau, J. & Trochon, P. Evolution en fréquence et en intensité de l'infestation des moules par *Mytilicola intestinalis* Steuer dans le bassin Marennes-Oléron pour la période 1960-1963. *Cult. Mar.* **14,** 12–13 (1964).
131. Morgan, E. Synecology in soft sediment bivalves: the influence of parasites, physiological processes, and environmental stressors on health and disease. **PhD,** (University College Cork, 2013).
132. Mortensen, S., Sælemyr, L., Skår, C. K., Bodvin, T. & Jelmert, A. *The surveillance and control*

*programme for bonamiosis and marteiliosis in European flat oysters,* Ostrea edulis*, and blue mussels,* Mytilus *sp. in Norway in 2015*. *Rapport Fra Havfroskingen* **23–2016,** (2015).

133. Munford, J. G., DaRos, L. & Strada, R. A study on the mass mortality of mussels in the Laguna Veneta. *J. World Maricul. Soc.* **12,** 186–199 (1981).
134. Murray, P. J. The occurrence of *Mytilicola intestinalis* Steuer on the coasts of counties Galway and Clare. *Ir. Nat. J.* **17,** 198–199 (1972).
135. Newell, G. E. XLVII The marine fauna of Whitstable. *Ann. Mag. Nat. Hist.* **12,** 321–350 (1954).
136. Orlando, E. Heterochromosomes in *Mytilicola intestinalis* Steuer (Copepoda). *Genetica* **44,** 244–248 (1973).
137. Pascual, C. & Quintana, R. Incidencia del *Mytilicola intestinalis* sobre el contenido en carotenos del mejillón cultivado. *Alimentaria* **209,** 41–43 (1990).
138. Paul, J. D. The incidence and effects of *Mytilicola intestinalis* in *Mytilus edulis* from the Rías of Galicia, North West Spain. *Aquaculture* **31,** 1–10 (1983).
139. Pesta, O. Die Metamorphose von *Mytilicola intestinalis* Steuer. *Z. wiss. Zool.* **88,** 78–98 (1907).
140. Picoche, C. *et al.* Towards the determination of *Mytilus edulis* food preferences using the dynamic energy budget (DEB) theory. *PLoS One* **9,** e109796 (2014).
141. Pogoda, B., Jungblut, S., Buck, B. H. & Hagen, W. Infestation of oysters and mussels by mytilicolid copepods: differences between natural coastal habitants and two offshore cultivation sites in the German Bight. *J. Appl. Ichthyol.* **28,** 756–757 (2012).
142. Polk, P. Some observations on the Crustacean fauna of the Sluice-dock (Bassin de Chasse) of Ostend. *Bijdr. tot Kennis der Mariene Fauna van Belgische Kust* **39,** 1–8 (1963).
143. Prinz, K., Kelly, T. C., O'Riordan, R. M. & Culloty, S. C. Occurrence of macroparasites in four common intertidal molluscs on the south coast of Ireland. *Mar. Biodivers. Rec.* **3,** (2010).
144. Rayyan, A., Photis, G. & Chintiroglou, C. C. Metazoan parasite species in cultured mussel *Mytilus galloprovincialis* in the Thermaikos Gulf (North Aegean Sea, Greece). *Dis. Aquat. Organ.* **58,** 55–62 (2004).
145. Rementeria, A. *et al.* Assessment of health status of oysters (*Crassostrea gigas*) exposed to environmentally relevant concentrations of Ag and Cu in brackish waters. *J. Sea Res.* **130,** 229–238 (2017).
146. Rementeria Ugalde, A. Understanding the impact of silver as an emerging contaminant in the Ibaizabal and Gironde estuaries. *Geochemistry* **PhD,** (Université de Bordeaux, 2016).
147. Robert, S. *et al. Réseau national d'observation de la moule bleue* Mytilus edulis *MYTILOBS / Campagne 2015*. **Final Report,** (2016).
148. Robledo, J. A. F., Cacares-Martinez, J. & Figueras, A. *Mytilicola intestinalis* and *Proctoeces maculatus* in mussel (*Mytilus galloprovincialis* Lmk.) beds in Spain. *Bull. Eur. Ass. Fish Pathol.* **14,** 89–94 (1994).
149. Robledo, J. A. F., Santarém, M. M. & Figueras, A. Parasite loads of rafted blue mussels (*Mytilus galloprovincialis*) in Spain with special reference to the copepod, *Mytilicola intestinalis*. *Aquaculture* **127,** 287–302 (1994).
150. Robledo, J. A. F., Santarém, M. M., González, P. & Figueras, A. Seasonal variations in the biochemical composition of the serum of *Mytilus galloprovincialis* Lmk. and its relationship to the reproductive cycle and parasitic load. *Aquaculture* **133,** 311–322 (1995).
151. Santarém, M. M., Robledo, J. A. F. & Figueras, A. Seasonal changes in hemocytes and serum defense factors in the blue mussel *Mytilus galloprovincialis*. *Dis. Aquat. Organ.* **18,** 217–222 (1994).
152. Socoro, N. M. Organo X (Organo de Bellonci) en larvas de *Mytilicola intestinalis* Steuer; Copepoda, Crustacea. *Bol. R. Soc. Esp. Hist. Nat.* **79,** 115–128 (1981).
153. Steuer, A. *Mytilicola intestinalis* n. gen. n. sp. aus dem Darme von *Mytilus galloprovincialis* Lam. *Zool. Anz.* **25,** 635–637 (1902).
154. Steuer, A. *Mytilicola intestinalis* n. gen. n. sp. *Arb. Zool. Inst., Univ. Wien* **15,** 1–46 (1905).
155. Stock, J. H. Parasiet veroorzaakt massasterfte onder mossels. *Zeepaard* **10,** 87–89 (1950).
156. Stock, J. H. On Copepoda associated with Dutch molluscs. *Basteria* **29,** 65–71 (1965).
157. Stock, J. H. Copepoda (Crustacea) associated with commercial and non-commercial Bivalvia in the East Scheldt, the Netherlands. *Bijdr. tot Dierkd.* **63,** 61–64 (1993).
158. Theisen, B. F. *Mytlilicola intestinalis* Steuer, en parasitisk Copepod ny for den danske fauna. *Flora og Fauna* **70,** 35–39 (1964).
159. Theisen, B. F. *Mytilicola intestinalis* Steuer in Danish waters. *ICES Counc. Meet. Shellfish Comm.* **103,** 1–4 (1964).

160. Theisen, B. F. *Mytilicola intestinalis* Steuer in Danish waters 1964-1965. *Meddelelser fra Danmarks Fisk. og Havundersøgelser* **4,** 327–337 (1966).
161. Theisen, B. F. *Mytilicola intestinalis* Steuer and the condition of its host *Mytilus edulis* L. *Ophelia* **27,** 77–86 (1987).
162. Vaysièrre, A. in *Les Bouches du Rhône. Encyclopédie départmental.* (eds. Caillol, H. & Vaysièrre, A.) **3 partie,** 278 pp (1914).
163. Vilela, H. & Manuela Correira Monteiro, M. Présence de Copépodes chez *Mytilus edulis* L. *ICES, C. M.* **87,** (1958).
164. Villalba, A., Mourelle, S. G., Carballal, M. J. & López, C. Symbionts and diseases of farmed mussels *Mytilus galloprovincialis* throughout the culture process in the Rías of Galicia (NW Spain). *Dis. Aquat. Organ.* **31,** 127–139 (1997).
165. Watermann, B. *et al.* Histopathological lesions of molluscs in the harbour of Norderney, Lower Saxony, North Sea (Germany). *Helgol. Mar. Res.* **62,** 167–175 (2008).
166. Waugh, G. D. The occurrence of *Mytilicola intestinalis* (Steuer) on the east coast of England. *J. Anim. Ecol.* **23,** 364–367 (1954).
167. Wickstead, J. A new record of *Mytilicola intestinalis* Steuer, a parasitic copepod of mussels. *Nature* **185,** 258 (1960).
168. Williams, C. S. The parasitism of young mussels by *Mytilicola intestinalis*. *J. Nat. Hist.* **1,** 299–301 (1967).
169. Williams, C. S. The influence of *Polydora ciliata* (Jonst.) on the degree of parasitism of *Mytilus edulis* L. by *Mytilicola intestinalis* Steuer. *J. Anim. Ecol.* **37,** 709–712 (1968).
170. Williams, C. S. The life history of *Mytilicola intestinalis* Steuer. *J. Cons. Int. Pour l'Exploitation la Mer* **32,** 419–428 (1969).
171. Zens, M. Der Einfluss von Parasiten auf Vitalität und Bestandsentwicklung der Miesmuschel (*Mytilus edulis* L.). (1999).
172. Zorita, I. *et al.* Evaluation of the use of bioaccumulation and biological effects tools in caged mussels, within the European Water Framework Directive. *Chem. Ecol.* **31,** 432–445 (2015).
173. Castagné, M. & Le Dantec, J. Le gisement huitrier de l'Adour. *Sci. Pêche* **107,** 1–5 (1962).

## Reference list for *Mytilicola orientalis*

Data from papers cited in this list underlie plotting of the *Mytilicola orientalis* distributions in Fig. 2, Supplementary Fig. S2, and Online Resource 2.


1. Bernard, F. R. Incidence and effect of the copepod *Mytilicola orientalis* Mori in the Pacific oyster of British Columbia. *Fish. Res. Bd. Can. MS Rept.* **993,** 14 (1968).
2. Bradley, W. & Siebert, A. E. J. Infection of *Ostrea lurida* and *Mytilus edulis* by the parasitic copepod *Mytilicola orientalis* in San Francisco Bay, California. *The Veliger* **21,** 131–134 (1978).
3. Carballal, M. J., Iglesias, D., Santamarina, J., Ferro-Soto, B. & Villalba, A. Parasites and pathologic conditions of the cockle *Cerastoderma edule* populations of the coast of Galicia (NW Spain). *J. Invert. Path.* **78,** 87–97 (2001).
4. Chew, K. K., Sparks, A. K. & Katkansky, S. C. First record of *Mytilicola orientalis* Mori in the California mussel *Mytilus californianus* Conrad. *J. Fish. Res. Bd. Can.* **21,** 205–207 (1964).
5. Chew, K. K., Sparks, A. K. & Katkansky, S. C. Preliminary results on the seasonal size distribution of *Mytilicola orientalis* and the effect of this parasite on the condition of the Pacific oyster, *Crassostrea gigas*. *J. Fish. Res. Bd. Can.* **22,** 1099–1101 (1965).
6. Chew, K. K., Sparks, A. K., Katkansky, S. C. & Hughes, D. Preliminary observations on the seasonal size distribution of *Mytilicola orientalis* Mori in the Pacific oyster, *Crassostrea gigas* (Thunberg) at Humboldt Bay, California, and Yaquina Bay, Oregon. *Proc. Natl. Shellfish Assoc.* **55,** 1–8 (1964).
7. Clanzig, S. Invertébrés d'introduction récente dans les lagunes méditerranéennes du Languedoc-Roussillon (France). *Bull. la Société Zool. Fr.* **114,** 151–152 (1989).
8. Comps, M. & Pichot, Y. *Situation zoosanitaire des coquillages en Mediterranée (1985-1987). Rapports internes de la Direction des Ressources Vivantes de l'IFREMER* (1989).
9. De Grave, S., Xie, Q. & Casey, D. The intensity of infestation by the intestinal copepod, *Mytilicola orientalis*, does not affect the condition of Pacific oysters (*Crassostrea gigas*). *Bull. Eur. Assoc. Fish Pathol.* **15,** 129–131 (1995).
10. Deslous-Paoli, J.-M. *Mytilicola orientalis* Mori, *Crassostrea gigas* Thunberg's parasite, in the basin of Marennes-Oléron: impact on the condition and the biochemical composition of oysters during rearing. *Int. Counc. Explor. Sea* **29,** 1–15 (1981).
11. Elsner, N. O., Jacobsen, S., Thieltges, D. W. & Reise, K. Alien parasitic copepods in mussels and oysters of the Wadden Sea. *Helgol. Mar. Res.* **65,** 299–307 (2010).
12. Farley, C. A., Wolf, P. H. & Elston, R. A. A long-term study of 'microcell' disease in oysters with a description of a new genus, *Mikrocystos* (g. n.), and two new species, *Mikrocystos mackini* (sp. n.) and *Mikrocystos roughleyi* (sp. n.). *Fish. Bull.* **86,** 581–593 (1988).
13. Francisco, C. J., Hermida, M. A. & Santos, M. J. Parasites and symbionts from *Mytilus galloprovincialis* (Lamark, 1819) (Bivalves: Mytilidae) of the Aveiro Estuary Portugal. *J. Parasitol.* **96,** 200–205 (2010).
14. Friedman, C. S., Brown, H. M., Ewing, T. W., Griffin, F. J. & Cherr, G. N. Pilot study of the Olympia oyster *Ostrea conchaphila* in the San Francisco Bay estuary: description and distribution of diseases. *Dis. Aquat. Organ.* **65,** 1–8 (2005).
15. Goater, C. P. & Weber, A. E. Factors affecting the distribution and abundance of *Mytilicola orientalis* (Copepoda) in the mussel, *Mytilus trossulus*, in Barkley Sound, BC. *J. Shellfish Res.* **15,** 681–684 (1996).
16. Goedknegt, M. A. Pacific oysters and parasites: Species invasions and their impact on parasite-host interactions. **PhD,** (VU University Amsterdam, 2017).
17. Goedknegt, M. A. *et al.* Spillover but no spillback of two invasive parasitic copepods from invasive Pacific oysters (*Crassostrea gigas*) to native bivalve hosts. *Biol. Invasions* **19,** 365–379 (2017).
18. His, E. Oberservations préliminaires sur la présence de *Mytilicola orientalis* Mori (1935) chez *Crassostrea gigas* Thunberg dans le bassin d'Arcachon. *Bull. Soc. Géol. Amis du Muséum du Havre* **LXIV,** 7–8 (1977).
19. His, E. Observations relatives à l'infestation de *Crassostrea gigas* Thunberg par le copépode parasite *Mytilicola orientalis* Mori dans le Bassin d'Arcachon. *Cons. Int. Explor. mer, coquillages benthos C.M.* **K:33,** 8 (1977).
20. His, E. Mytilicolides et Myicolides parasites des lamellibranches d'intérêt commercial du Bassin d'Arcachon. *Haliotis* **8,** 99–102 (1979).

21. His, E., Tige, G. & Rabouin, M. A. *Mytilicola orientalis* Mori: son action sur les huîtres du bassin d'Arcachon au cours de l'été et de l'automne 1977. *Cons. Int. Pour l'Exploitation la Mer* **K:14,** (1978).
22. Ho, J.-S. Origin and dispersal of *Mytilus edulis* in Japan deduced from its present status of copepod parasitism. *Publ. Seto Mar. Biol. Lab.* **25,** 293–313 (1980).
23. Ho, J.-S. & Kim, I.-H. Copepod parasites of gastropods from Korea. *J. Zool.* **35,** 240–255 (1992).
24. Holmes, J. M. C. & Minchin, D. Two exotic copepods imported into Ireland with the Pacific oyster *Crassostrea gigas* (Thunberg). *Ir. Nat. J.* **25,** 17–20 (1995).
25. Jeung, H.-D. *et al.* Histopathologic observation of the Mediterranean mussel, *Mytilus galloprovincialis* (Lamarck, 1819) during a spawning season. *Ocean Polar Res.* **36,** 121–134 (2014).
26. Katkansky, S. C., Sparks, A. K. & Chew, K. K. Distribution and effects of the endoparasitic copepod, *Mytilicola orientalis*, on the Pacific oyster, *Crassostrea gigas*, on the Pacific coast. *Proc. Natl. Shellfish. Assoc.* **57,** (1967).
27. Katkansky, S. C. & Warner, R. W. On the unusual occurrence of the copepod *Mytilicola orientalis* in the digestive diverticulae of the Pacific oyster, *Crassostrea gigas*. *J. Invert. Path.* **12,** 475–476 (1968).
28. Katkansky, S. C. & Warner, R. W. Pacific oyster disease and mortality studies in California, May 1966–June 1972. *Calif. Dept. Fish Game, Mar. Res. Tech. Rept.* **25,** 51 (1974).
29. Kim, I. Poicilostomatoid copepods associated with bivalves in Korea and their distribution. *Zool. Stud.* **43,** 187–192 (2004).
30. Kim, I.-H. & Sato, S. A review of copepods associated with bivalves in Japan, with description of two new species (Crustacea, Copepoda, Cyclopoida). *Bull. Tohoku Univ. Museum* **9,** 1–22 (2010).
31. Kovačić, I., Pavičić-Hamer, D., Pfannkuchen, M. & Usich, M. *Mytilus galloprovincialis* (Lamarck, 1819) as host of *Mytilicola orientalis* (Mori, 1935) in the northern Adriatic Sea: presence and effect. *Aquac. Int.* **25,** 211–221 (2016).
32. Le Pennec, G. & Le Pennec, M. Histopathological damages caused by the copepod *Mytilicola orientalis* in the oyster *Crassostrea gigas* from the Bay of Brest (Britanny, France). *Haliotis* **32,** 73–80 (2003).
33. Meyer, G. R. *et al.* Health Status of Olympia Oysters (*Ostrea lurida*) in British Columbia, Canada. *J. Shellfish Res.* **29,** 181–185 (2010).
34. Minchin, D., Duggan, C. B., Holmes, J. M. C. & Neiland, S. Introductions of exotic species associated with Pacific oyster transfers from France to Ireland. *Int. Counc. Explor. Sea Maric. Comm.* **F:27,** (1993).
35. Minchin, D. & Nunn, J. Rapid assessment of marinas for invasive alien species in Northern Ireland. *North. Irel. Environ. Agency Res. Dev. Ser.* **13/06,** (2013).
36. Moore, J. D., Juhasz, C. I. & Robbins, T. T. A histopathology survey of California oysters. *Calif. Fish Game* **97,** 63–83 (2011).
37. Mori, T. *Mytilicola orientalis*, a new species of parasitic Copepoda. *Zool. Mag. Tokyo* **47,** 687–689, pls. 1–3 (1935).
38. Nagasawa, K. & Nitta, M. Rediscovery of *Mytilicola orientalis* (Copepoda: Mytilicolidae) from wild Pacific oysters in *Crassostrea gigas* in Japan. *Biogeography* **16,** (2014).
39. Odlaug, T. O. The effect of the copepod, *Mytilicola orientalis* upon the Olympia oyster, *Ostrea lurida*. *Trans. Am. microsc. Soc.* **65,** 311–317 (1946).
40. Pogoda, B., Jungblut, S., Buck, B. H. & Hagen, W. Infestation of oysters and mussels by mytilicolid copepods: differences between natural coastal habitants and two offshore cultivation sites in the German Bight. *J. Appl. Ichthyol.* **28,** 756–757 (2012).
41. Sparks, A. K. Some preliminary observations on the infection and pathological effect of the parasitic copepod, *Mytilicola orientalis* Mori, in the Pacific oyster (*Crassostrea gigas* (Thunberg)) on the west coast of the United States. *Int. Counc. Explor. Sea Shellfish Com.* **139,** 1–9 (1962).
42. Sparks, A. K., Chew, K. K. & Katkansky, S. C. *A report on the incidence and pathology of* Mytilicola *in oysters for 1963*. (University of Washington, 1964).
43. Steele, S. & Mulcahy, M. F. Gametogenesis of the oyster *Crassostrea gigas* in southern Ireland. *J. Mar. Biol. Ass. U.K.* **79,** 673–686 (1999).
44. Stock, J. H. Copepoda (Crustacea) associated with commercial and non-commercial Bivalvia in the East Scheldt, the Netherlands. *Bijdr. tot Dierkd.* **63,** 61–64 (1993).
45. Stock, J. H. Een tweede soort *Mytilicola* in Nederland. *Zeepaard* **53,** 41–42 (1993).

46. Suh, H.-L. & Choi, S.-D. Two copepods (Crustacea) parasitic on the blue mussels, *Mytilus galloprovincialis*, from the Yongsan River estuary in Korea. *Bull. Korean Fish. Soc.* **23,** 137–140 (1990).
47. Yamazaki, H. About a species of parasite in the alimentary tract of oysters. *Suishi Dayori* **9,** 86–89 (1950).
